# Oxygen Vacancy Distribution in Yttrium-Doped Ceria from ^89^Y–^89^Y Correlations via Dynamic Nuclear Polarization Solid-State NMR

**DOI:** 10.1021/acs.jpclett.1c00221

**Published:** 2021-03-17

**Authors:** Daniel Jardón-Álvarez, Nitzan Kahn, Lothar Houben, Michal Leskes

**Affiliations:** † Department of Materials and Interfaces, Weizmann Institute of Science, Rehovot 76100, Israel; ‡ Department of Chemical Research Support, Weizmann Institute of Science, Rehovot 76100, Israel

## Abstract

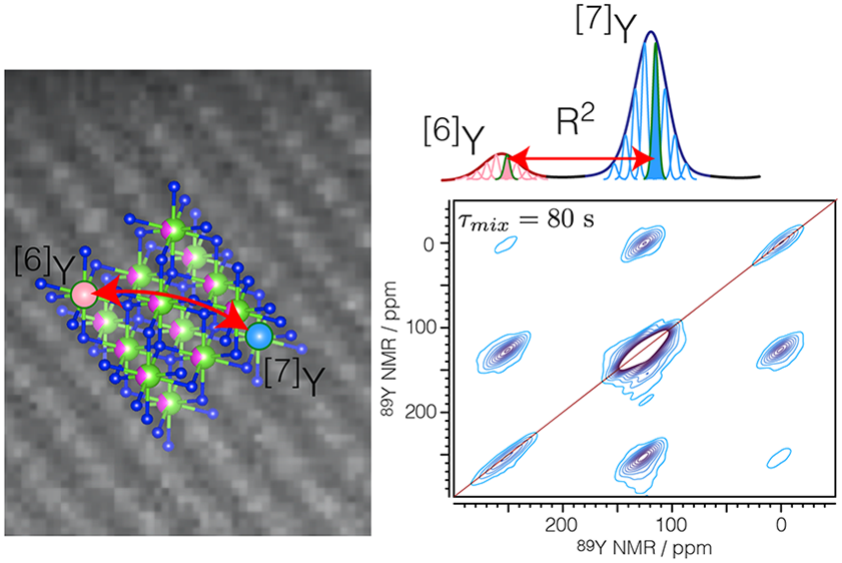

Comprehending the oxygen vacancy distribution in oxide ion conductors requires structural insights over various length scales: from the local coordination preferences to the possible formation of agglomerates comprising a large number of vacancies. In Y-doped ceria, ^89^Y NMR enables differentiation of yttrium sites by quantification of the oxygen vacancies in their first coordination sphere. Because of the extremely low sensitivity of ^89^Y, longer-range information was so far not available from NMR. Herein, we utilize metal ion-based dynamic nuclear polarization, where polarization from Gd(III) dopants provides large sensitivity enhancements homogeneously throughout the bulk of the sample. This enables following ^89^Y–^89^Y homonuclear dipolar correlations and probing the local distribution of yttrium sites, which show no evidence of the formation of oxygen vacancy rich regions. The presented approach can provide valuable structural insights for designing oxide ion conductors.

Solid oxide fuel cells^1^ are one of the most promising options for satisfying our high energy demands because of the potential of high efficiency paired with low environmental impact.^2−4^ One of the key aspects for the success of these devices is improving the oxygen ion conductivity in the solid electrolyte. Some of the most effective materials for these applications are based on ceria, CeO_2_. Doping ceria with trivalent cations, like Y^3+^, creates oxygen vacancies (OV), thus increasing the ionic conductivity. Understanding the structural changes that lead to enhanced properties is crucial to systematically design new materials with improved characteristics.

The distribution of OV within the lattice is critical for the ionic conductivity,^5^ but characterizing homogeneity over various length scales with varying OV concentration is challenging, and proposed structural descriptions are still under debate. For instance, based on diffraction data, the existence of nanodomains of Y_2_O_3_ within CeO_2_ has been proposed^6^ and is supported by other experimental findings.^7−10^ On the other hand, nuclear magnetic resonance (NMR) data suggested the preferred formation of yttrium pairs, but the absence of larger clusters,^11^ in line with a structural model proposed by Małecka et al.^12,13^ for Yb^3+^-doped ceria. Efforts to obtain a full understanding of the structure need to focus on connecting long-range structural properties with an understanding at the atomic scale.^14,15^

Solid-state NMR spectroscopy is very powerful for characterizing the local atomic structure of materials. In the context of doped ceria, NMR has been used for determining local preferences of cation coordination^11,16−18^ and has also been exploited for studying ion mobility by temperature-dependent relaxation studies.^18−23^ However, correlating information on nearest, or next nearest, neighbors obtained from NMR parameters with larger structural motifs is more challenging.^24−27^

Here we exploit through-space homonuclear dipolar couplings under the rotational resonance (R^2^) condition^28,29^ among ^89^Y spins from different sites to obtain intermediate-range structural information directly linked to the short-range structure readily obtained from NMR spectra. The weak dipolar couplings among yttrium spins make the R^2^ condition extremely selective, which paired with large inhomogeneous broadenings present in these samples, strongly reduces the probability of finding a pair of spins matching the R^2^ condition. Consequently, mapping the magnetization exchange reflects the probability of finding one site within a medium-range distance environment of a second site.

A major difficulty in studying yttrium-doped ceria (YDC) via NMR lies in the low sensitivity of the NMR-active nuclei. Common strategies for sensitivity enhancement consist of introducing paramagnetic agents to shorten relaxation times or isotopic (^17^O) enrichment. We have recently shown how metal ion-based dynamic nuclear polarization (MIDNP) under magic angle spinning (MAS) can lead to significant sensitivity enhancements of low-sensitivity nuclei in inorganic materials.^30,31^ The possibility of measuring the bulk of inorganic samples differentiates this approach from conventional exogeneous DNP,^32^ which has been applied on similar materials to study their surface properties.^33−36^

In this work we exploit the sensitivity gain obtained from MIDNP for characterizing Yttrium distributions in YDC. We prepared various samples following the coprecipitation route^37^ of composition Ce_1–*x*–*y*_Y_*x*_Gd_*y*_O_2–*x*/2–*y*/2_ with *x* = 0.1, 0.25, and 0.4 and *y* = 0.001 and 0.005 (hereon labeled *x*Y*y*GC, with *x* and *y* given in %). The effect of Gd^3+^, which is introduced as polarizing agent, on the OV distribution will be assumed to be negligible. Figure 1 shows the DNP-enhanced ^89^Y spectra. The obtained enhancements represent a time savings of 4 orders of magnitude. The field sweep profile obtained for 10Y01GC (Figure S6) indicates that the DNP enhancement is mediated by the solid effect mechanism. Nuclear spin relaxation in these samples is governed by paramagnetic relaxation enhancement^38^ from the introduced gadolinium (see Table S2, Figure S8, and discussion in the Supporting Information). Because spin diffusion is not expected to be efficient for any of the present nuclei, the large DNP enhancements can be related to direct polarization, in accordance with our previous results.^31^ Consequently, DNP will yield homogeneous enhancements throughout the entire bulk of the sample, and the results will have quantitative value. In agreement, no qualitative changes in the line shape are observed in the enhanced spectra. The large enhancements further allowed measurement of natural abundance ^17^O NMR (Figure S12).

**Figure 1:**
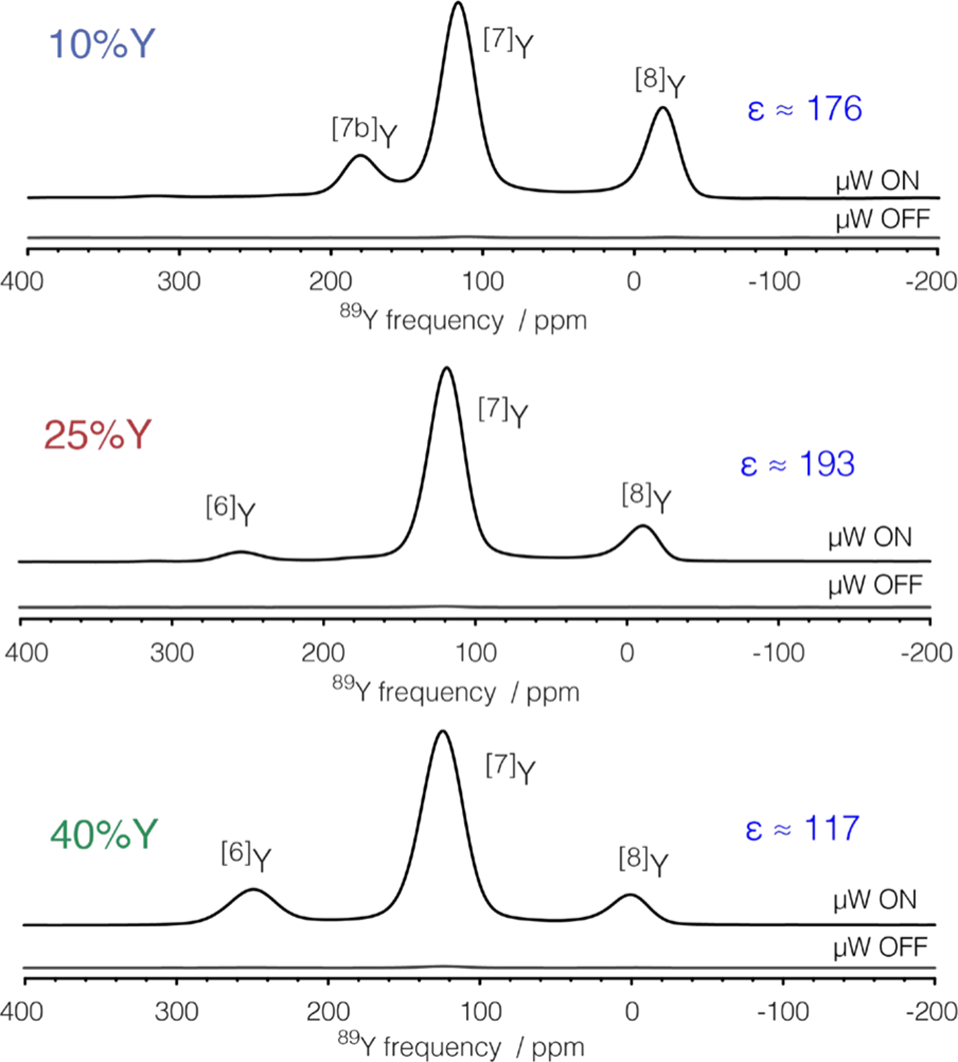
^89^Y MAS NMR Hahn echo spectra with and without microwaves (μW) irradiation of 10Y01GC, 25Y01GC, and 40Y01GC, from top to bottom. The spinning speed (ν_R_) was 8 kHz for 10Y01GC and 25Y01GC and 10 kHz for 40Y01GC. The recycle delay was 1910, 2270, and 1570 s, respectively, approximately representing the duration of buildup of 95% of the magnetization. Spectra under μW irradiation were acquired with four scans.

The obtained spectra show three main peaks assigned to 8-, 7- and 6-coordinated yttrium sites.^11,16^ The decrease in coordination number represents the presence of oxygen vacancies. Isotropic chemical shifts, line widths, and relative intensities are given in Table S1 and are in agreement with previously reported values.^11,16^ The relative intensities of the various peaks reflect a deviation from randomness of the coordination of OV, which prefer to coordinate at the trivalent yttrium cation sites.^11,16^ At the lowest Y concentration we observe an additional peak (^[7b]^Y) at 180 ppm, which has been attributed to a distinct 7-coordinated environment.^16^ In all samples most of the yttrium is in 7-coordinated sites, evidencing that the yttrium ions are distributed among ceria instead of forming clusters of C-type structure.^6^ In the latter case one would expect dominant signals from 6-coordinated yttrium^39,40^ growing in intensity with increasing concentration. The large peak broadening is inhomogeneous in nature and reflects a distribution of local environments, underlining that even low concentrations of yttrium doping distort the entire ceria fluorite structure.

The increased sensitivity enabled measurements of ^89^Y–^89^Y correlation spectra^41^ (Figure 2). To our knowledge, homonuclear correlation experiments among nuclei with such weak gyromagnetic ratio have not been reported previously. Using the rotational resonance (R^2^) approach^28,29^ has a series of advantages for this system over other common schemes.^42^ Because of the weak couplings a prohibitively large amount of radio frequency pulses would be necessary with most recoupling sequences. Further, the short transverse (in the order of a few milliseconds) and very long longitudinal relaxation times (hundreds of seconds) require dipolar evolution while the magnetization is stored along the longitudinal direction.

**Figure 2:**
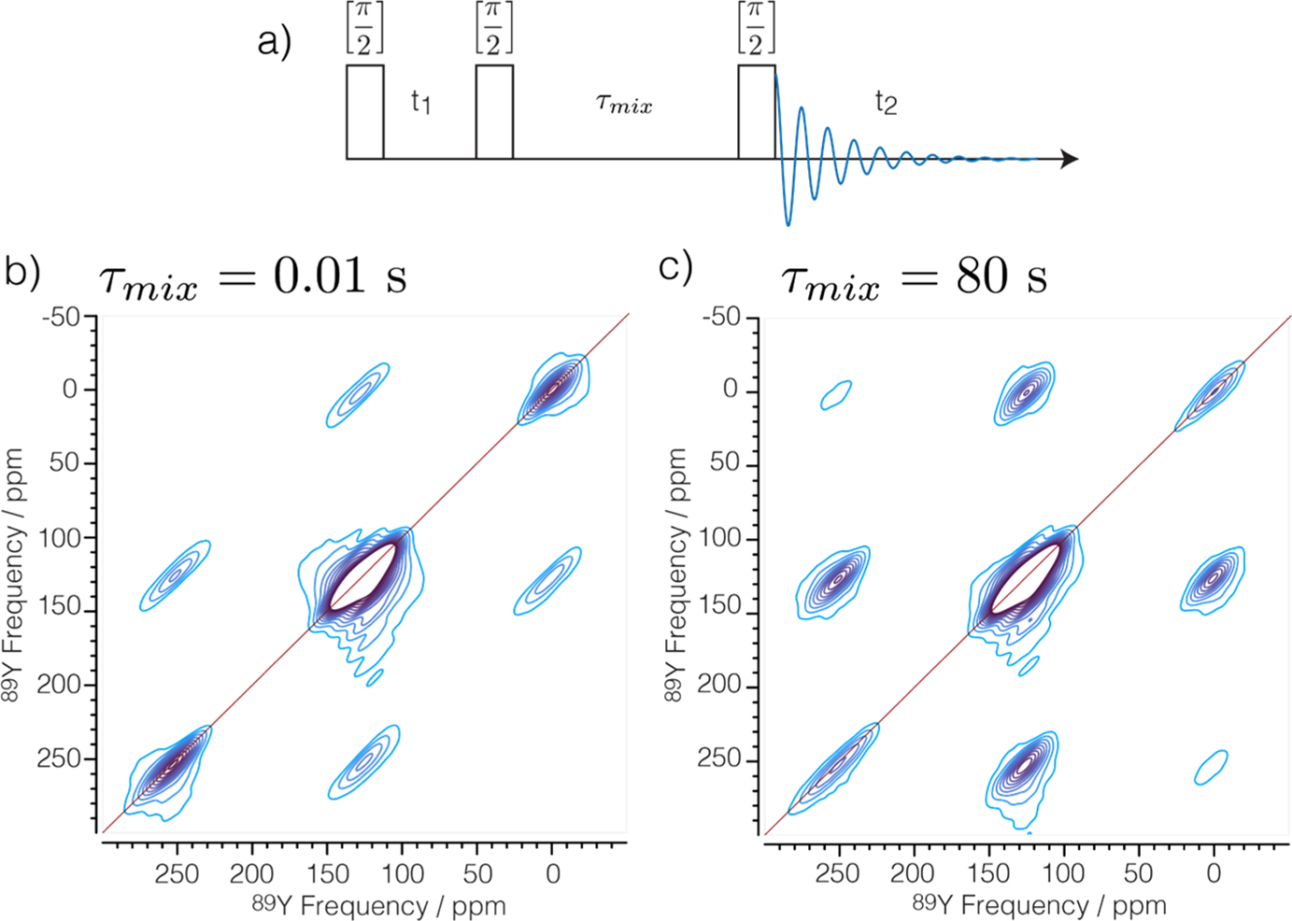
Rotational resonance ^89^Y 2D MAS DNP NMR spectra of 40Y01GC obtained with the pulse sequence shown in panel a using a mixing time of 0.01 (b) and 80 s (c). The spinning speed (ν_R_) was set to 2470 Hz, equivalent to the separation between the ^[7]^Y peak and both the ^[6]^Y and ^[8]^Y peaks. A recycle delay of 70 s and 40 equally spaced increments in the indirect dimension (*t*_1_) were used, and 8 scans were acquired. The total experiment time was 12.5 and 26.3 h, respectively. Twenty equally spaced contours are plotted from 2 to 20% of the maximum intensity.

At the R^2^ condition ( where *n* is a small integer,  the MAS frequency, and  the isotropic chemical shift),^29^ dipolar couplings are not averaged out by MAS. Figure 2 shows 2D ^89^Y–^89^Y correlation experiments of 40Y01GC. Because each additional OV has approximately the same effect on the isotropic chemical shift, the R^2^ condition can be fulfilled for all three sites simultaneously. The off-diagonal peaks observed after a mixing time of 10 ms are attributed to spinning sidebands (see Figures S7 and S10). After 80 s the relative intensity of the cross peaks is considerably larger and can be attributed to ^89^Y homonuclear dipolar couplings.

An in-depth study of the exchange of Zeeman order as a function of the mixing time was carried out with 1D spectra after selective inversion of both the ^[6]^Y and ^[8]^Y signals (Figure S11). To account for longitudinal relaxation during the mixing time, an additional set of analogue control experiments was performed without the inversion step. The ratio between both experiments shows the evolution of the magnetization due to the dipolar couplings. For a proper normalization the effect of the spinning sidebands also needs to be considered. The normalized intensities are shown in Figure 3. We notice two interesting features which we will analyze in the following: first, surprisingly long mixing times are required for reaching a steady state, and second, differential characteristic times are observed among samples, but not for different sites within individual samples.

**Figure 3:**
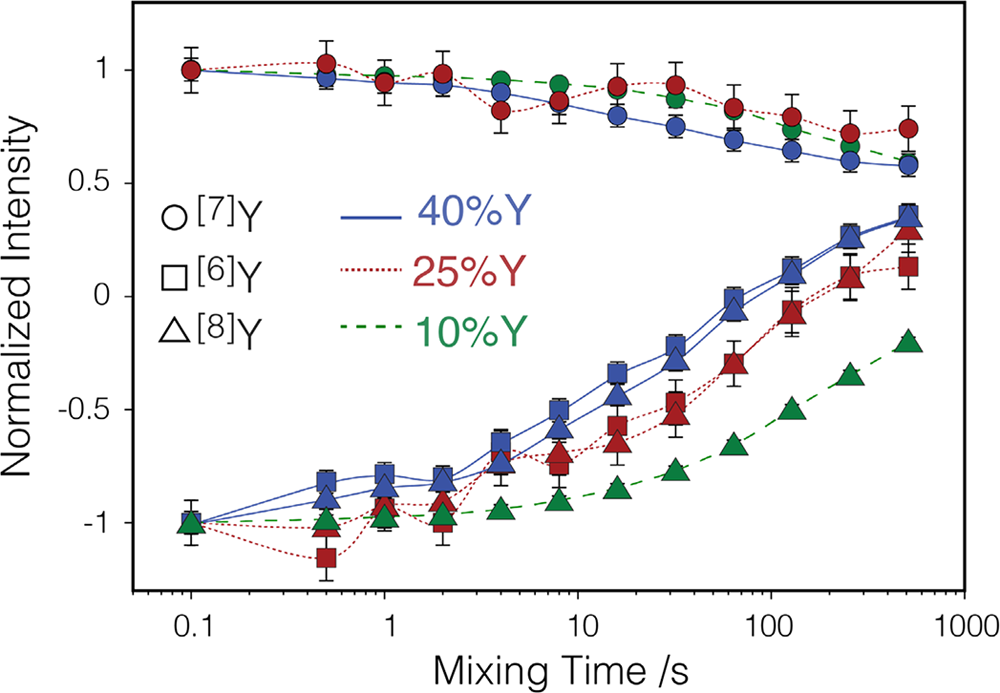
Evolution of the normalized integrated signal intensity of the ^[6]^Y (squares), ^[7]^Y (circles), and ^[8]^Y (triangles) sites in R^2^ correlation experiments. The sequence shown in Figure 2a was used, but with fixed *t*_1_ time of 187.27, 192.3, and 202.4 μs for 10Y01GC (dashed green line), 25Y01GC (dotted red lines), and 40Y01GC (solid blue lines), respectively, creating an initial  state. The data was normalized with respect to an additional set of experiments with *t*_1_ of 1 μs (creating an initial  state). The spinning speed ν_R_ was 2.67, 2.60, and 2.47 kHz, respectively. A recycle delay of 360 s was used, and 8 scans were acquired.

The largest possible dipolar coupling of about 5 Hz represents a few orders of magnitude shorter time scales compared to the measured magnetization exchange. Two distinct mechanisms can cause a slowing of exchange, zero quantum relaxation times (*T*_2ZQ_), and large inhomogeneous broadenings.^29,43^ The exchange of Zeeman order is slowed down when the ZQ decoherence rate is larger than the coupling strength.^29^ When the source of transverse decay of two coupled nuclei is uncorrelated, their individual transverse relaxation time, *T*_2_, gives a good estimate of *T*_2ZQ_. Here the main source of *T*_2_ is the Gd ions. For two yttrium nuclei at close proximity the local fluctuating fields originating from distant gadolinium ions will be very similar. Thus, this mechanism will not modulate the dipolar flip-flop operator, *I*_*+*_*I*_*–*_, and therefore would not be an efficient source of *T*_2ZQ_.^44,45^ Consequently, *T*_2ZQ_ is likely to slow the magnetization exchange only for distant yttrium pairs, and the short (few milliseconds) *T*_2_ measured in these samples are not a proper estimate of *T*_2ZQ_ for relevant coupled pairs, which is likely to be much larger. Small (±3 Hz) experimental spinning instabilities might be an additional source contributing to *T*_2ZQ_, although its effect is unlikely to dominate the exchange curve. Second, the very weak couplings lead to an extremely sensitive R^2^ matching condition. The homogeneous broadening of the ^89^Y signal is about 1 order of magnitude smaller than the inhomogeneous (from approximately 35 to 500 Hz, see line shape in the 2D maps^46^ and *T*_2_ analysis in Figure S9). Consequently, the probability of two close proximity spins satisfying the rotational resonance condition is reduced,^43^ and the mean distance between spins matching the R^2^ condition increases.

A series of numerical simulations to tentatively reproduce possible scenarios were performed with the SPINEVOLUTION program^47^ for small spin systems (ranging from single pairs to up to 11 coupled spins). The most relevant results for isolated spin pairs are summarized in Figure 4, and further results including larger spin systems are given in the Supporting Information. The already mentioned effect of a frequency offset from the R^2^ matching condition and of *T*_2ZQ_ on the buildup curves can be seen in Figure 4. This discussion evidences a complicated correlation between coupling strength, *T*_2ZQ_, and the magnetization exchange, making determination of exact distance distributions among sites unfeasible. Nonetheless, the measured curves carry valuable information: they are indicative of the probability of occurrence of the proper condition between spin pairs.

**Figure 4:**
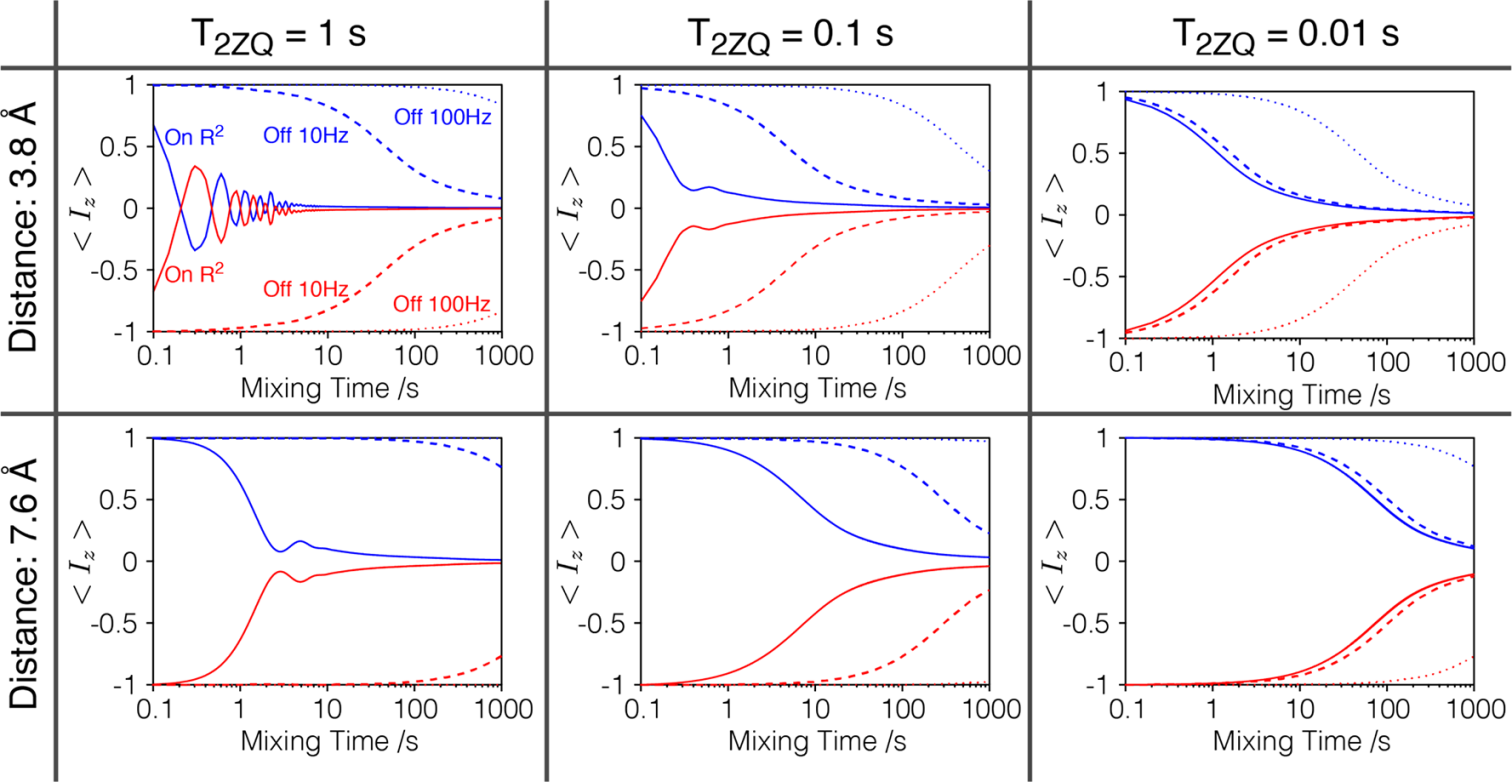
MAS NMR simulations showing the evolution of the *z* component of the magnetization in a ^89^Y–^89^Y rotational resonance correlation experiment for a single pair of ^89^Y spins, with an isotropic chemical shift difference of 2470 Hz, after averaging over 233 crystallite orientations. An initial state with opposite sign of longitudinal magnetization for both nuclei was chosen. Distances of 3.8 and 7.6 Å and relaxation times *T*_2ZQ_ of 0.01, 0.1, and 1 s were considered. The solid lines show a case where the R^2^ condition was matched exactly, an offset of 10 Hz is shown with dashed lines and an offset of 100 Hz with the dotted lines. The spinning speed ν_R_ was set to 2470 Hz, matching the rotational resonance condition. Further details are given in the Supporting Information.

Accurate estimation of *T*_2ZQ_ would be a challenging task. However, as noted above, for a pair of spins in close proximity ZQ relaxation due to the paramagnetic center will have low efficiency. Further, because of the lack of strong NMR interactions in these samples, it is unlikely that relaxation mechanisms from nuclear dipolar couplings will be efficient sources of relaxation. Consequently, we would expect long *T*_2ZQ_ relaxation times with the upper limit being *T*_1_, which is in the order of hundreds of seconds in these samples (see the Supporting Information). For a *T*_2ZQ_ of 1 s, the rotational resonance condition is already extremely selective; the slow observed experimental Zeeman exchange could be explained within this scenario by an increased probability of finding a matching pair with increasing distance, accompanied by a decreasing coupling strength. However, we need to keep in mind that increasing distances can also lead to a reduced *T*_2ZQ_. Additionally, slow polarization transfer can occur between nuclei in close proximity, but this requires short *T*_2ZQ_ and large frequency offsets. Interestingly, for large offsets, shortening of *T*_2ZQ_ can result in a faster polarization transfer. Combination of these findings with the experimental observations shown in Figure 3 points toward the presence of a broad distribution of the various relevant parameters, distances, relaxation times, and frequency offsets. In agreement with this, the measured curves show a distinctly flatter shape compared to the simulations, a consequence of the distribution of magnetization exchange rates over various orders of magnitude from spin pairs in different conditions.

Finally, our experimental results (Figure 3) show that with increasing concentration the exchange of Zeeman order becomes faster. This is in line with a larger probability of encountering Y-pairs fulfilling the R^2^ condition, as percolation of yttrium throughout the structure advances. The clear distinction between different samples indicates that yttrium is spread in a homogeneous manner throughout the lattice instead of forming segregated, OV rich clusters. In 25Y01GC and 40Y01GC, where ^[6]^Y, ^[7]^Y, and ^[8]^Y sites are present, no distinct behavior is observed for the ^[6]^Y and ^[8]^Y sites within experimental errors. This indicates that there is no preferential proximity between ^[7]^Y and ^[6]^Y or ^[8]^Y and points toward an absence of clustering of OV, which would increase the probability of ^[6]^Y and ^[7]^Y sites being in closer proximity. In agreement, electron microscopy measurements (see the Supporting Information) on these samples did not show the presence of clustering within the resolution limit of about 1 nm.

Our results point toward a gradual transition from distorted fluorite to distorted C-type structure.^48^ This is in agreement with the continuous bulk model,^13^ which does not exclude superstructural motifs,^49^ observed in the diffraction data of the highly doped sample (Figure S1). In this process, however, we do not observe any evidence of a deviation from a random distribution of yttrium and cerium. Nevertheless, generalization should be taken with care, as the synthesis route plays a critical role in the structural properties.^5^

Here we have presented an alternative approach for studying medium range structural properties via NMR. Our experimental results do not show evidence of OV clustering in yttrium-doped ceria. This experimental study has been possible by taking advantage of a series of adverse NMR properties, namely, a low gyromagnetic ratio, long T_1_ relaxation time, and strong inhomogeneously broadened lines. While each of them individually contributes to difficult acquisition of NMR spectra, we have shown that in combination they can be exploited for gaining new structural information. The main requirement being a sufficiently large signal per transient, which was accomplished by MIDNP.

## Supporting Information Available

Details on sample preparation; X-ray powder diffraction; EPR measurements; EM and EDX measurements; details on NMR measurements, as well as DNP field sweep profiles, analysis of spectra and relaxation data, spectra obtained at rotational resonance condition, and natural abundance ^17^O NMR spectrum; NMR simulations (PDF)
