# Supplementary material for: Oxygen Vacancy Distribution in Yttrium-Doped Ceria from 89Y–89Y Correlations via Dynamic Nuclear Polarization Solid-State NMR
Source: J Phys Chem Lett. 2021 Mar 17;12(11):2964–9. doi: 10.1021/acs.jpclett.1c00221 (PMC8006133; doi:10.1021/acs.jpclett.1c00221)
Supplement: Supplementary file 1 — jz1c00221_si_001.pdf [file jz1c00221_si_001.pdf]

Supporting Information

Oxygen Vacancies Distribution in Yttrium Doped Ceria from ^{89}Y-^{89}Y Correlations via Dynamic Nuclear Polarization Solid State NMR

Daniel Jardón-Álvarez[(1)], Nitzan Kahn[(1)], Lothar Houben[(2)] and Michal Leskes[(1)]*

[(1)]Department of Materials and Interfaces, Weizmann Institute of Science, Rehovot, 76100, Israel

[(2)]Department of Chemical Research Support, Weizmann Institute of Science, Rehovot, 76100, Israel

List of Contents

1. Sample Preparation
2. X-Ray Powder Diffraction
3. EPR Measurements
4. EM and EDX
5. NMR Measurements
    a. DNP field sweep profiles
    b. Spectral deconvolution
    c. Relaxation
    d. Rotational resonance
    e. Natural abundance ^{17}O
6. NMR Simulations

1. Sample Preparation

Samples were prepared following the co-precipitation route described in ref.[1] by weighting stoichiometric quantities of the precursors $Y(NO_3)_3 \cdot 6H_2O$ (Strem Chemicals, 99.9%), $Gd(NO_3)_3 \cdot 6H_2O$ (Strem Chemicals, 99.9%) and $Ce(NO_3)_3 \cdot 6H_2O$ (Strem Chemicals, 99.9%). Precipitation was achieved with $(NH_4)_2CO_3$ (Aldrich, ACS reagent grade).

2. X-Ray Powder Diffraction

Formation of the desired structure was confirmed with X-ray powder diffraction (Figure S1). Measurements were performed on a TTRAX-III Rigaku diffractometer equipped with a rotating Cu anode operating at 50 kV and 200 mA. Phase analysis was

done with the software JADE 2010. All prepared samples were high purity materials with less than 1 % impurities. Incorporation of yttrium cations in the lattice was confirmed by a decrease in the unit cell parameter of the cubic fluorite structure (space group Fm-3m). The obtained unit cell lattice parameters, accounting only for the peaks for the fluorite structure (vide infra) are 5.4095 ± 0.0001 Å, for 10Y05GdC, 5.4100 ± 0.0001 Å, for 10Y01GdC, 5.4016 ± 0.0001 Å, for 25Y01GC and 5.3918 ± 0.0001 Å for 40Y01GC.

In the sample with largest yttrium content additional peaks were observed (see inset in Figure S1). This behavior has already been reported within this concentration range and is attributed to superstructure peaks indicating an ordering of the OV positions creating a repetition unit larger than the fluorite unit cell[2,3]. This can be understood as a consequence of a phase transition from fluorite to C-type. The peak positions are in good agreement with the space group Ia-3, the relative intensity of the superstructure peaks and the pure fluorite peaks depend on the yttrium content. The position of the superstructure peaks are in agreement with the reported values for 43.75 % yttrium by by Coduri et al.[3]

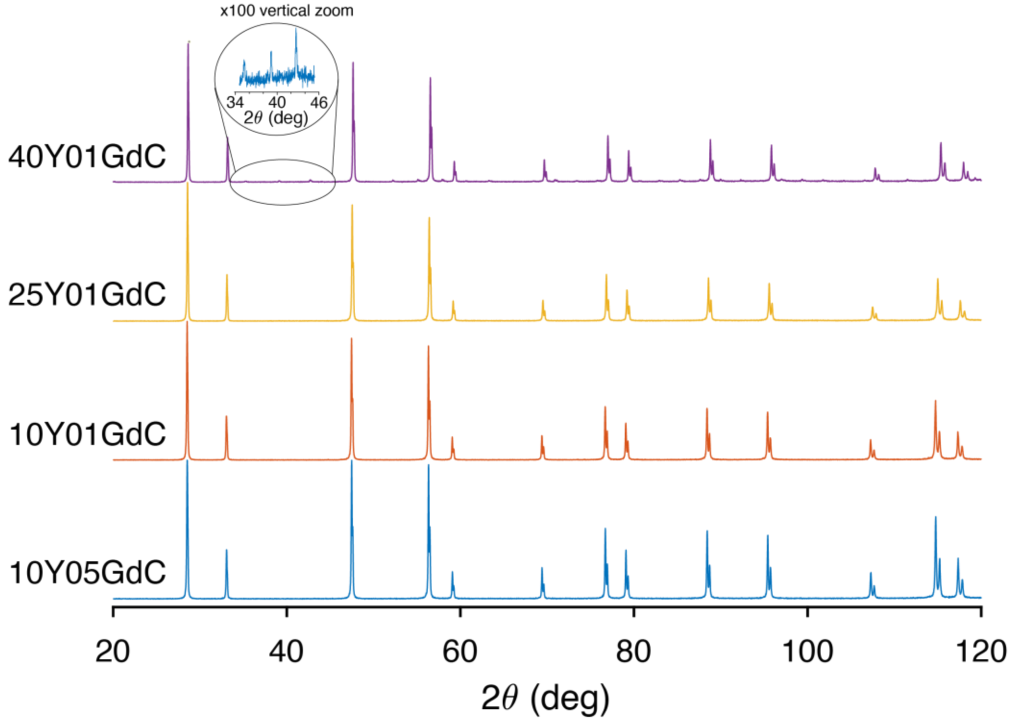

Figure S1. X-ray powder diffraction pattern of 10Y05GdC, 10Y01GdC, 25Y01GC and 40Y01GC, from bottom to top.

3. EM and EDX

High-resolution scanning transmission electron microscopy (STEM) images and energy dispersive X-ray spectroscopy (EDS) maps were recorded in a double aberration-corrected Themis Z microscope (Thermo Fisher Scientific Electron Microscopy Solutions, Hillsboro, USA) equipped with a high-brightness FEG at an accelerating voltage of 200 kV. High angle annular dark-field images (HAADF) images were recorded with a Fischione Model 3000 detector with a semi-convergence angle of 30 mrad, a probe current of 40 pA, and an inner collection angle of 70.0 mrad. EDS hyperspectral data were obtained with a Super-X G2 four-segment SDD detector with a probe semi-convergence angle of 21 mrad, a beam current of approximately 200 pA, a pixel dwell time of 20 µs and a total recording time of typically 10 minutes. Quantitative maps were calculated with the Velox software (Thermo Fisher Scientific Electron Microscopy Solutions, Hillsboro, USA), through background subtraction and spectrum deconvolution.

Figure S2 shows STEM images of 25Y01GC. The contrast is in agreement with a single phase material with random site occupancy on the cation sites as indicated in the model in Figure S2c. EDS maps for all different yttrium concentrations are shown in Figure S3. In neither sample clustering of yttrium could be observed at a scale larger than 1 nm. The specular appearance of the maps is due to the statistical fluctuations which limits the sensitivity for clustering to volumes of about 1 nm in size. The determined atomic percentages of Ce: 26.0 ± 4.1 at %, Y: 2.8 ± 0.5 at %, O: 71.1 ± 8.3 at %; Ce: 23.1 ± 3.4 at %, Y: 7.9 ± 1.3 at %, O: 69.0 ± 8.3 at %; and Ce: 17.8 ± 2.5 at %, Y:

14.3 ± 2.2 at %, O: 67.9 ± 6.5 at % for 10Y01GdC, 25Y01GC and 40Y01GC, respectively are in reasonable agreement with the expected values from the nominal compositions.

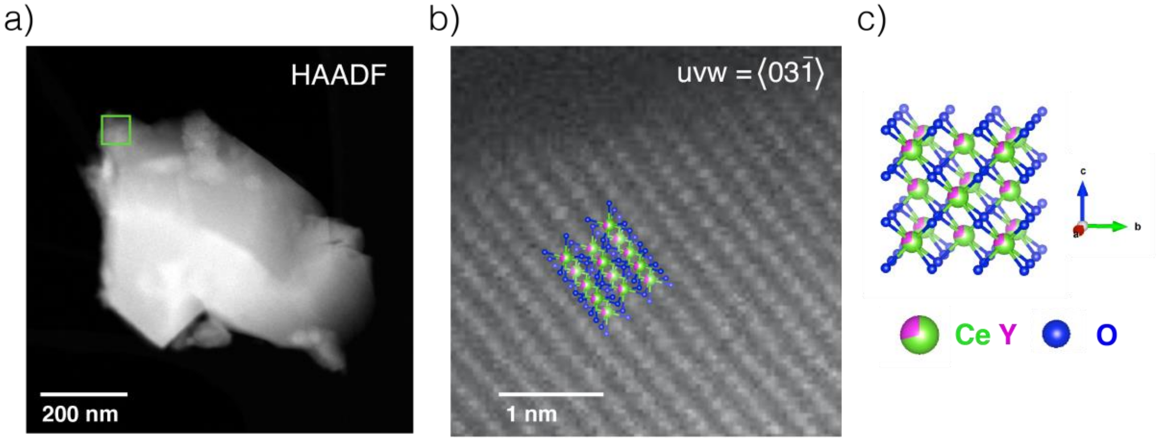

Figure S2. STEM images of 25Y01GC. (a) Low magnification HAADF image of a micrometer size single crystal. (b) Magnified region indicated by the rectangle marker in (a) showing the crystal edge in a ⟨03$\bar{1}$⟩ viewing direction of the cubic fluorite structure of the alloyed cerium oxide. (c) Model of the fluorite structure with random cation site occupancy.

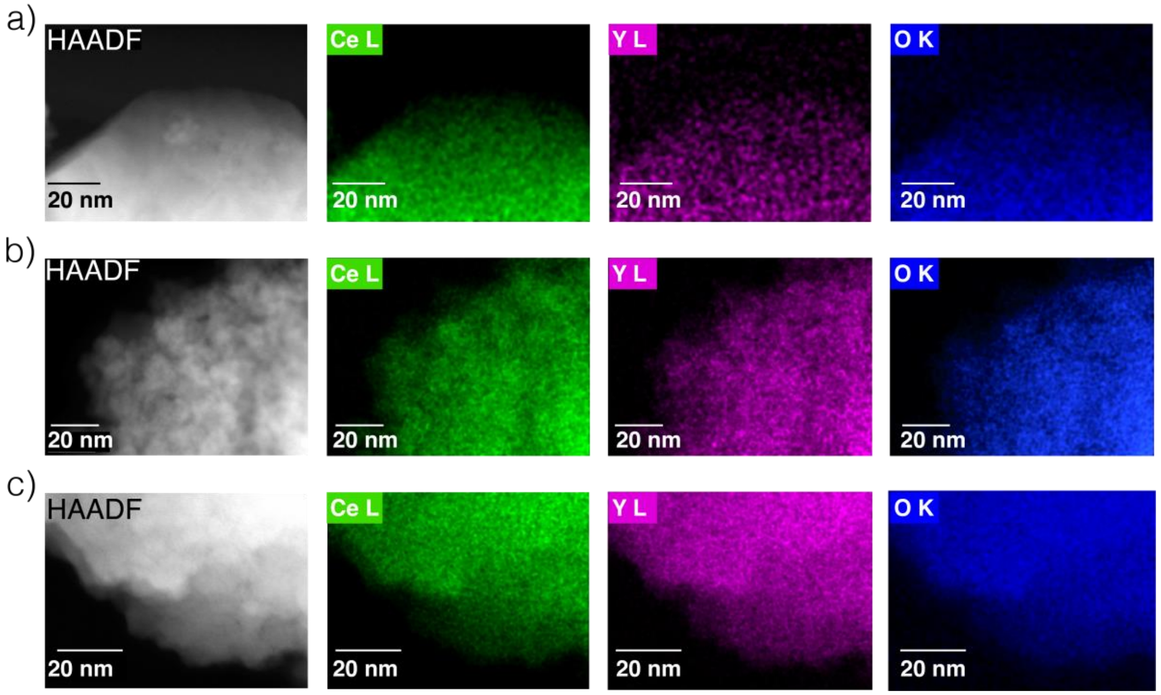

Figure S3. STEM images, corresponding EDS elemental maps and compositional analysis of $Ce_{0.999-x}Y_xGd_{0.001}O_{1.9995-x/2}$ with three nominal alloy compositions. (a) x = 0.1, (b) x = 0.25, and (c) x = 0.4. The elemental EDS maps were obtained after proper background subtraction.

4. EPR Measurements

Continuous wave (CW) electron paramagnetic resonance (EPR) spectra were acquired at 50 K at X- (9.4 GHz) and Q-band (34 GHz) on a Bruker ELEXYS E-580 spectrometer. The obtained X-band results are shown in Figure S4. The Q-band results are shown in Figure S5. Increasing the gadolinium content to 0.5% causes significant broadening of the EPR line.

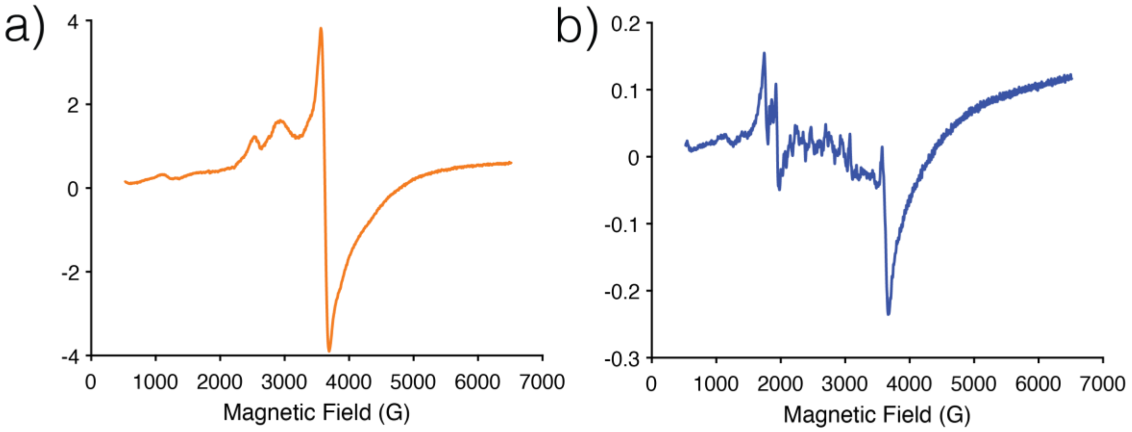

Figure S4. X-Band EPR spectra of (a) 10Y05GdC and (b) 10Y01GdC obtained at 50 K.

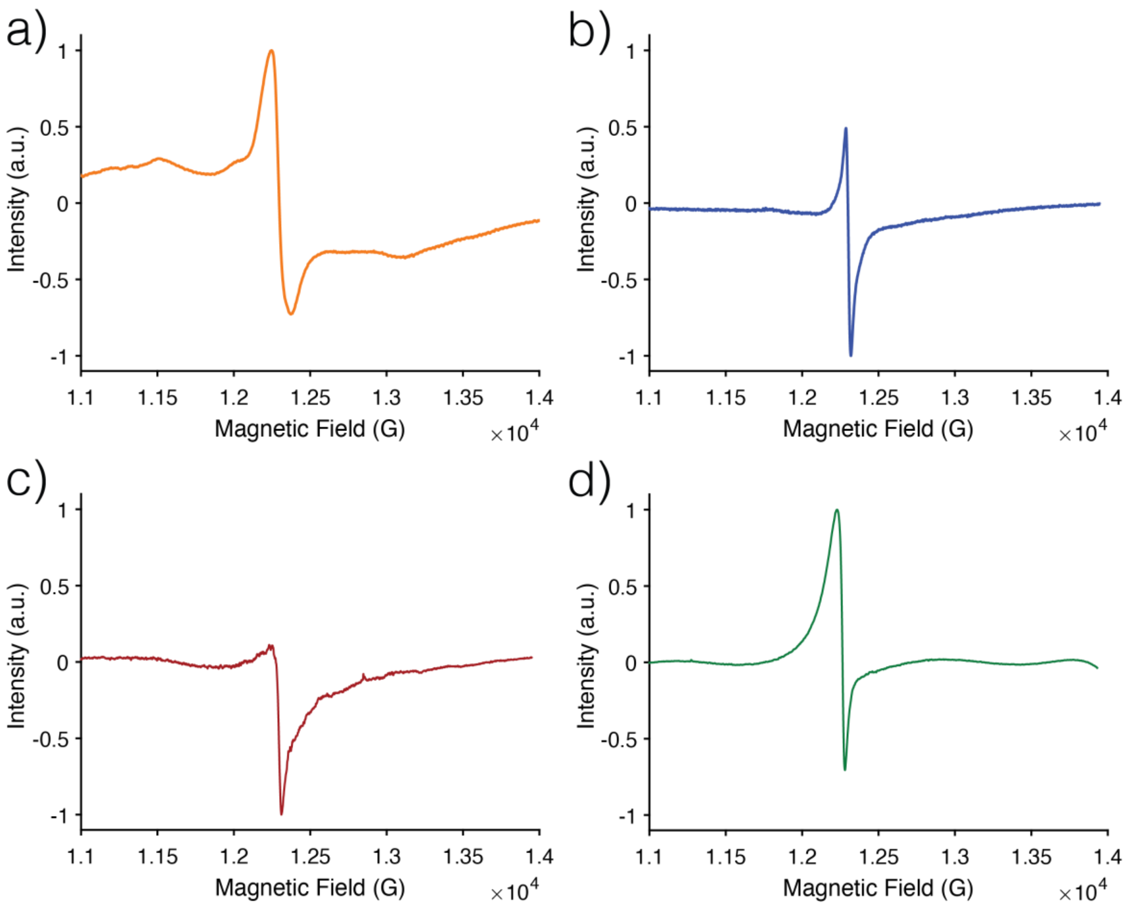

Figure S5. Q-Band EPR spectra of (a) 10Y05GdC, (b) 10Y01GdC, (c) 25Y01GC and (d) 40Y01GC obtained at 50 K

5. NMR Measurements

All NMR measurements were performed on a Bruker 9.4 T Avance-Neo spectrometer equipped with a sweep coil and a 263 GHz gyrotron system. All measurements were done using a 3.2 mm double resonance LT- DNP probe at around 100 K. MAS rates were stable within ±3 Hz, the rates used in the rotational resonance experiments are given in the corresponding figure captions, all other measurements were performed at 8 kHz, unless otherwise specified. Spectra were obtained with the Hahn echo sequence using an echo delay of one rotor period. The rf-amplitudes used were 71.4 and 23.8 kHz, for ^{17}O and ^{89}Y,

respectively. ^{89}Y chemical shift was referenced to $Y(NO_3)_3 \cdot 6H_2O$ at room temperature at -53.2 ppm[4]. Build-up times, T_{BU}, under microwaves (µW) irradiation were determined using the saturation recovery sequence[5] and the measured data was fitted to a stretched exponential function:

$$M_z(t) = M_z(\infty) \cdot \left[1 - \exp\left[-\left(\frac{t}{T_{BU}}\right)^{\beta}\right]\right]. \tag{S1}$$

Transverse magnetization decay times, T_2, were determined using the Hahn echo sequence with variable echo delay and the measured data was fitted to a stretched exponential function:

$$M_{xy}(t) = M_{xy}(0) \cdot \exp\left[-\left(\frac{t}{T_2}\right)^{\beta}\right]. \tag{S2}$$

Processing of the NMR spectra was done with RMN 1.8.6[6], spectral deconvolution and CSA analysis were done with DMFIT[7].

a. DNP field sweep profiles

The DNP field sweep profiles obtained for Y10Gd01C and Y10Gd05C are shown in Figure S6. The profile of Y10Gd01C shows maximum positive and negative enhancements separated by twice the Larmor frequency, indicating that the solid effect is the dominating DNP mechanism. The broadening of the EPR lines caused by higher gadolinium content results in broader features of the DNP field sweep profile and a reduced enhancement factor of approximately 40, compared to 176 obtained with 0.1 % Gd. Since spin diffusion is not expected to be efficient for any of the present nuclei and nuclear spin relaxation in these samples is governed by paramagnetic relaxation enhancement[8] from the introduced gadolinium, the DNP enhancements arise from direct polarization from the gadolinium center. We have shown in previous work that the steady state enhancement for this scenario is independent of the distance between polarizing agent and nucleus[9].

The magnetic field which gave the maximum enhancement for sample Y10Gd01C was used for 25Y01GC and 40Y01GC without furhter optimization. Note that the enhancements are negative and a 180° phase correction on the µW ON spectra was applied for esthetic purposes in Figure 1 of the main document.

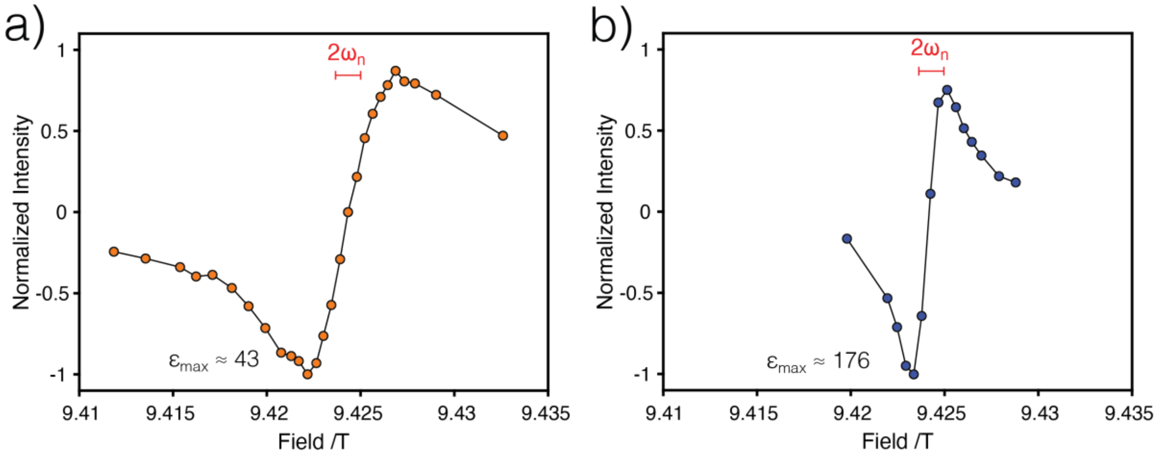

Figure S6. DNP sweep profiles for ^{89}Y in (a) 10Y05GdC and (b) 10Y01GdC.

b. Spectral deconvolution

Isotropic chemical shift (δ_{iso}), area and full width at half maximum (FWHM) obtained for the different yttrium sites after deconvolution of the ^{89}Y MAS NMR spectra are summarized in Table S1. The line broadenings increase with higher yttrium concentration and lower coordination number, indicating larger lattice distortions. Interestingly, the additional peak ($^{[7b]}Y$) observed in 10Y01GC, which has been attributed to a second 7-coordinated yttrium environment with differences in the second coordination sphere[10], was not present in the analogue sample with larger gadolinium content, 10Y05GC. At this point, parameters leading to its presence or absence are not understood. We also note that all peaks are strongly broadened compared to pristine Y_2O_3[11] (same is true for ^{17}O compared to CeO_2[12]). ^{89}Y chemical shift anisotropies were estimated from the spectrum of 40Y01GC obtained at a spinning speed of 3.6 kHz (Figure S7).

Y (mol %)	$^{[6]}$Y δ_{iso} (ppm)	Area (%)	FWHM (Hz)	$^{[7]}$Y δ_{iso} (ppm)	Area (%)	FWHM (Hz)	$^{[7b]}$Y δ_{iso} (ppm)	Area (%)	FWHM (Hz)	$^{[8]}$Y δ_{iso} (ppm)	Area (%)	FWHM (Hz)
10	315	0.24	228	116	60.78	345	180	12.53	326	-19	26.45	262
10*	-	0	-	135	80.60	501	-	0	-	2	19.40	356
25	255	4.64	558	119	80.34	500	178	1.38	570	-11	13.64	467
40	250	13.15	691	125	75.27	608	-	0	-	0	11.58	544

Table S1. Summary of the parametes obtained from deconvolution of the ^{89}Y spectra shown in Figure 1 and the spectrum obtained for 10Y05GC (spectrum not shown and indicated with an asterisk in table) assuming mixed Gaussian/Lorentzian line shapes.

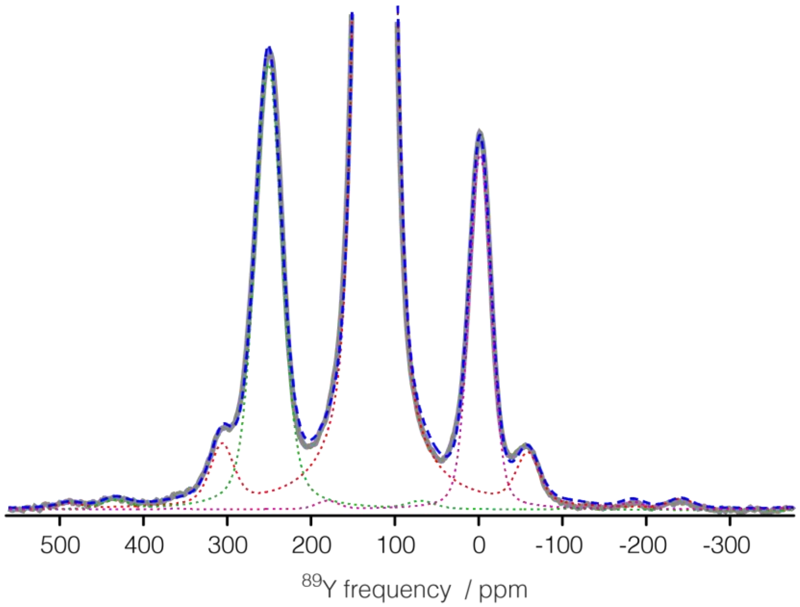

Figure S7. Thick gray line is the ^{89}Y MAS DNP NMR spectrum of 40Y01GC obtained at a spinning speed of 3.6 kHz. Best fit simulated spectrum (dashed blue line) was obtained by fitting the experimental spectrum with three sites $^{[6]}$Y (dotted green line), $^{[7]}$Y (dotted red line) and $^{[8]}$Y (dotted purple line) for estimating the chemical shift anisotropy. Good fit was obtained with an anisotropy of $\delta_{aniso} = \delta_{zz}$-$\delta_{iso}$ = 2.0 kHz for all three sites, while the asymmetry parameter was held constant at $\eta = 0$. While the uncertainty for $^{[7]}$Y is within 0.2 kHz, for $^{[6]}$Y and $^{[8]}$Y it is much larger, as only one spinning sideband can be distinguished at this spinning speed. These values are similar to the reported CSA in Y_2O_3.[13]

c. Relaxation

Longitudinal magnetization build-up times were obtained from fittings of the saturation recovery curves (Figure S8). The transverse magnetization decay of 25Y01GC was obtained from a Hahn echo experiment of variable echo delay (Figure S9). Due to the rigidity of the structure at 100 K, the weakness of the homonuclear couplings and the absence of heteronuclei, the main source of both longitudinal and transverse relaxation is paramagnetic relaxation enhancement caused by the gadolinium ions.

We note some variations in both the T_{BU} relaxation times and the ^{89}Y enhancements (Table S2) of samples with nominally the same dopant concentration. These deviations could be due to a reduced symmetry around the gadolinium center, following the increment in oxygen vacancies with increasing yttrium concentration[14]. Such scenario would lead to shorter T_{1e} and consequently smaller enhancement factors. Larger enhancements have been observed in the absence of yttrium, probably due to the conservation of high symmetry[15]. This reduced T_{1e} would also cause a shortening of nuclear T_1 according to PRE theory[16]. An alternative possible source of the observed variations could simply be related to small variations in the gadolinium concentration, which could result in a broadening of the EPR line.

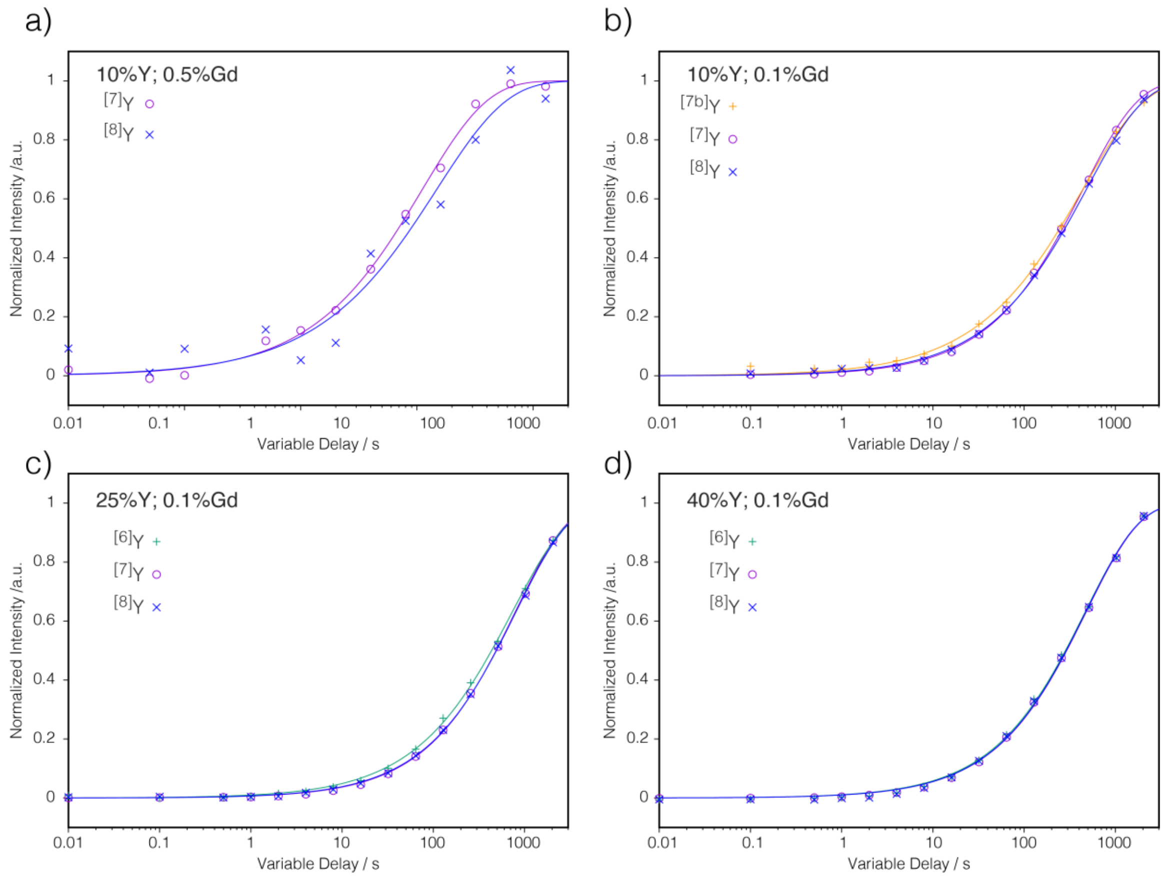

Figure S8. DNP buildup curves from integrated intensities of the ^{89}Y NMR signal obtained for the individual sites from a Hahn echo saturation recovery experiment. Solid curves are best fits obtained from fitting to equation S1. Intensities are normalized with respect to $M_z(\infty)$.

It should be highlighted that we do not observe differential T_1 relaxation times for different sites within a sample (Figure S8). Following the same rationale as for yttrium, one could also expect OV to preferentially coordinate on gadollinium[14]. Thus, the lack of differential relaxation can be understood as an additional indication of the absence of clustering between OV associated to Gd^{3+} with OV from Y^{3+}. This result is not in disagreement with the interpretation from EXAFS results by Deguchi et al.[17] who reported clustering of OV in yttrium doped ceria but lack of such clustering in the gadolinium analogue. They did not, however, perform a study with mixed dopants.

Gd Content		Y (mol %)	T_{BU} (s)	β	ε
mol %	mmol/L				
0.5	210	10	11.7 ± 0.5	0.80 ± 0.02	43
0.1	40	10	450 ± 20	0.70 ± 0.01	176
0.1	40	25	768 ± 40	0.77 ± 0.01	193
0.1	40	40	502 ± 30	0.73 ± 0.01	117

Table S2. ^{89}Y DNP buildup times T_{BU} obtained from fitting the integrated intensity of the sum of all sites to equation S1.

We estimated the homogeneous linewidth from the decay of the echo tops in a Hahn echo experiment with variable echo delay. As the exponential decay has a strongly stretched shape, we estimated the full width at half maximum (FWHM) of the homogeneous broadening from Fourier transform of a stretched exponential function with the values from the transverse magnetization decay.

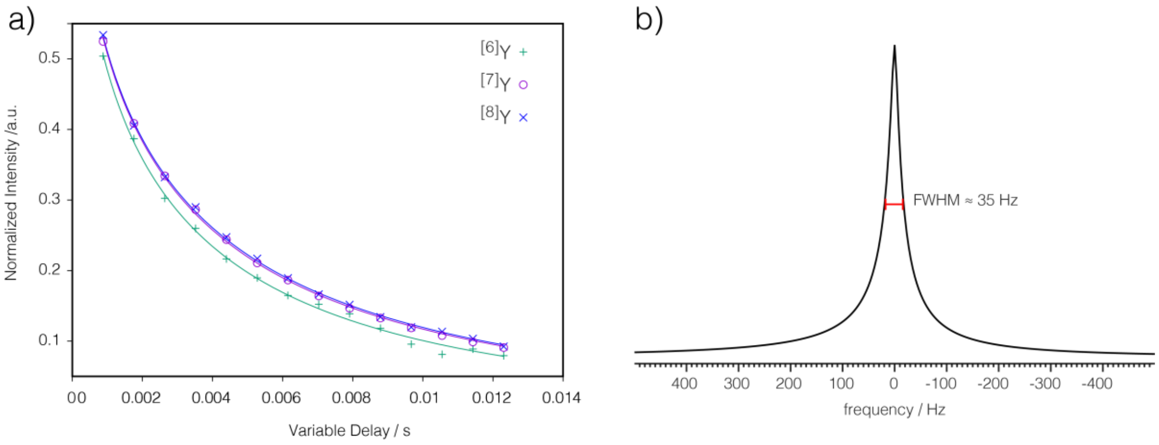

Figure S9. a) Transverse magnetization decay from integrated intensities of the ^{89}Y NMR signal of 25Y01GC obtained for the individual sites from a Hahn echo experiment of variable echo delay with an increment of 0.88 ms and at a spinning speed ν_R of 9.1 kHz. Solid curves are best fits obtained from fitting to a stretched exponential equation (equation (S2)) with fixed $\beta = 0.5$. Best fit transverse decay values of 1.89 ± 0.05 ms, 2.17 ± 0.03 ms and 2.21 ± 0.03 ms for $^{[6]}$Y, $^{[7]}$Y amd $^{[8]}$Y, respectively, were obtained. Intensities are normalized with respect to $M_{xy}(0)$. No significant variations of the transverse decay curves were observed as a funtion of the spinning speed (not shown here). b) Fourier transform of a stretched exponential function with $T_2 = 2$ ms and $\beta = 0.5$.

d. Rotational resonance

Figure S10 (a) shows an ^{89}Y 2D MAS DNP NMR correlation spectrum of 10Y01GC with a mixing time of 18 s. Additionally, 2D spectra with mixing times of 0.05, 1, 4 and 9 s were acquired and two selected cross sections are shown in Figure S10 (b). Distinction between spinning sidebands, constant in intensity, and dipolar cross peaks is evident. Cross-peaks between the $^{[8]}Y$ and $^{[7b]}Y$ sites could also be observed by choosing an appropriate spinning speed (not shown here).

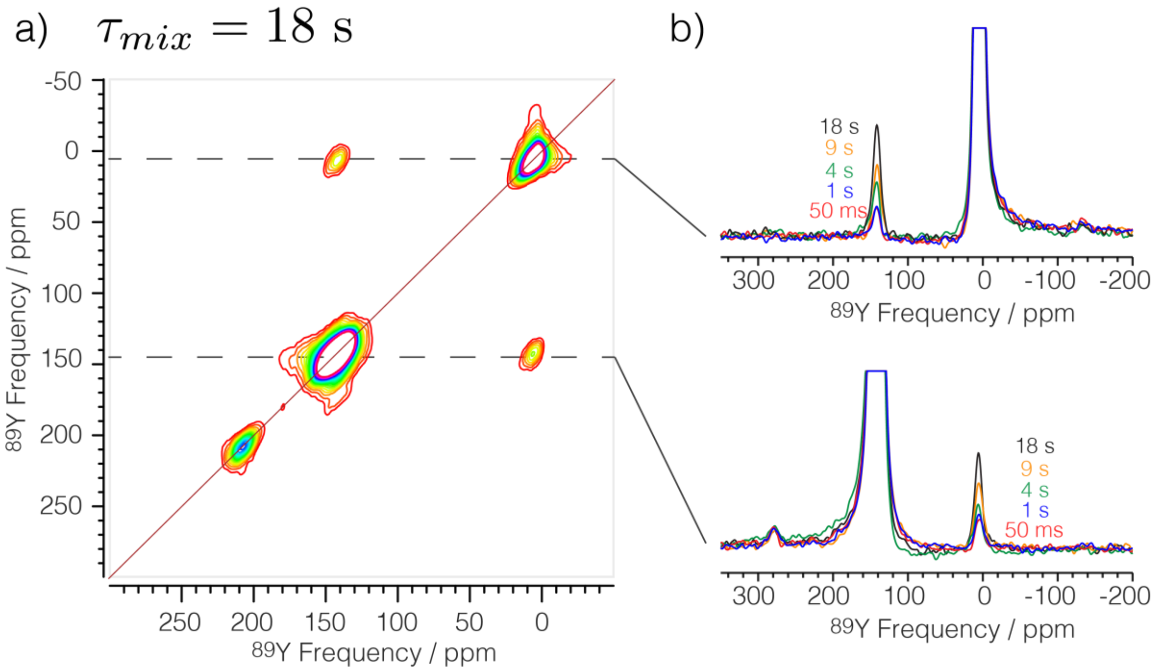

Figure S10. (a) Rotational resonance ^{89}Y 2D MAS DNP NMR spectrum of 10Y01GC using a mixing time of 18 s and at a spinning speed of 2680 Hz. (b) Selected cross sections of the 2D spectrum obtained with varying mixing times and normalized to the maximum intensity.

Evolution of the exchange of Zeeman order was mapped by inverting the magnetization of the $^{[6]}Y$ and $^{[8]}Y$ signals relative to the $^{[7]}Y$ signal, and storing it along the longitudinal axis for a variable time τ_{mix} while at the rotational resonance condition. Selective inversion of both signals was achieved by exploiting the fact that both peaks have approximately the same difference in isotropic chemical shift relative to $^{[7]}Y$, using the three pulse sequence, shown in Figure 2 of the main document, with a fixed t_1 time ensuring a ninety degree phase shift between signals. The small relative intensity of the overlapping spinning sidebands (Figure S7) does not impede good selectivity[18]. Spectra were acquired for 12

different logarithmically spaced mixing times between 0.1 and 512 s and the results are shown in Figure S11. In addition to the dipolar coupling induced modulation of the signal the intensity decays due to longitudinal relaxation. Note that in sample 25Y01GC the [6]Y and [8]Y peaks have a zero-crossing at clearly distinct times. This is not due to a distinct mean dipolar coupling strength to the [7]Y site, but rather an artifact of the presence of non-inverted spinning sidebands. The intensity of the [6]Y site in 25Y01GC is very low, and only slightly larger than the overlapping spinning sideband of the [7]Y peak. After accounting for the overlapping spinning sidebands, the intensities of the [6]Y and [8]Y sites cross zero after the same mixing time, within the experimental error (compare Figure 3 in main document).

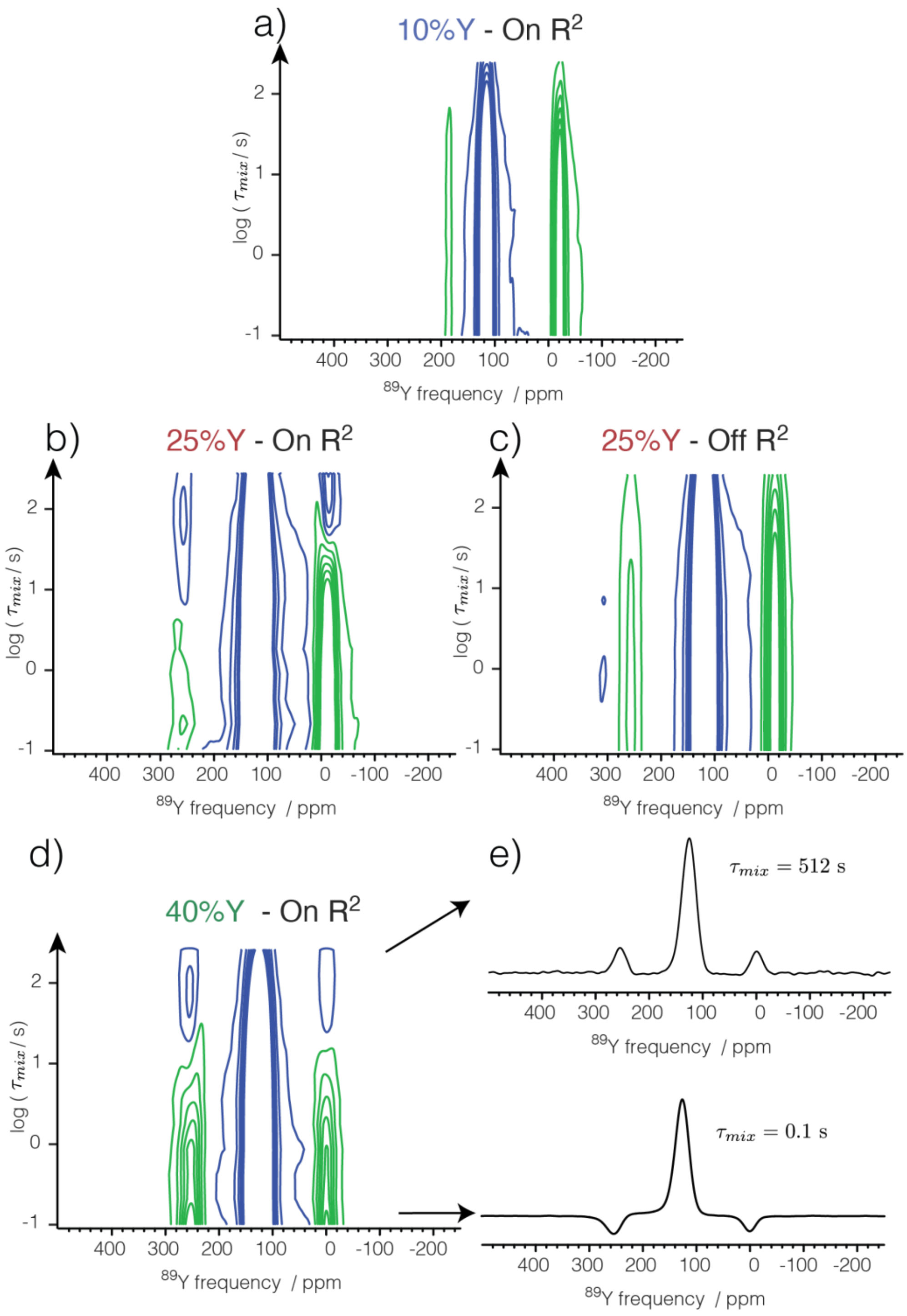

Figure S11. ^{89}Y MAS DNP NMR spectra obtained using the sequence shown in Figure 2a of the main document, with fixed values of t_1, to invert the magnetization of the $^{[6]}$Y and $^{[8]}$Y signals relative to the $^{[7]}$Y signal. Figures (a) - (d) show each 12 spectra linearly stacked, but obtained with logarithmically spaced variable mixing times between 0.1 and 512 s, along the vertical dimension. (a), (b) and (d) are the spectra for 10Y01GC, 25Y01GC and 40Y01GC, respectively, obtained at the rotational resonance condition (2.67, 2.6 and 2.47 kHz, respectively). (c) shows the evolution of 25Y01GC off the rotational resonance condition at 3.6 kHz. (e) shows two spectra of 40Y01GC obtained with shortest and longest mixing times. Green represents negative intensity, while blue positive.

e. Natural abundance ^{17}O DNP-NMR

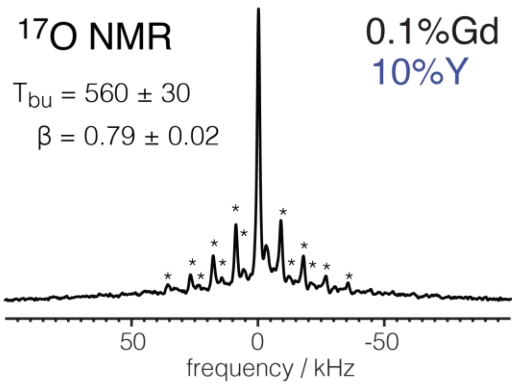

Figure S12. Natural abundance ^{17}O MAS DNP NMR spectrum of 10Y01GC. Obtained with 32 scans and a recycle delay of 450 s, giving a total experimental time of 4 hours. Spinning sidebands are marked with asterisks.

6. Rotational Resonance Simulations

In order to justify our interpretation of the experimental findings a series of simulations were performed using the SPINEVOLUTION program[19]. In these simulations we analyzed how the exchange of Zeeman order is affected by large inhomogeneous broadening and zero quantum relaxation T_{2ZQ}. Simulations reproduce ^{89}Y nuclear spins in an external magnetic field of 9.4 T under MAS. In order to reproduce a situation comparable to the experiments, differences in isotropic chemical shifts between groups of spins were set to $\Delta\omega_{iso}/2\pi = \Delta\nu_{iso} = 2470$ Hz and equal to the spinning speed ν_R, deviations from these values are explicitly stated in the figure captions. For polycrystalline models, a CSA of 2 kHz was used and the relative tensor orientation was chosen randomly for each individual spin. In order to observe the evolution of exchange of Zeeman order, an initial state with opposite sign of longitudinal magnetization was chosen for the different spin groups ($\rho(0) = \pm I_{zn}$). The obtained results are divided into two parts, summarized in the following paragraphs and corresponding figures. First, we focused on the effects of mismatch of the rotational resonance (R^2) condition and second, the effect of T_{2ZQ} was analyzed.

Under the R^2 condition, the longitudinal magnetization of two coupled spins will oscillate about their mean value with a frequency determined by the strength of the dipolar coupling due to the effect of the flip-flop operator I_+I_- (Figure S13b). Due to the angular dependence

of the dipolar coupling with respect to the external magnetic field, in a polycrystalline sample the distribution of coupling strengths leads to a dampening of the oscillation (Figure S13c). Deviations from the R^2 condition lead to an incomplete exchange of Zeeman order (Figure S14). The strictness of the condition becomes more severe as the coupling strength becomes weaker (Figure S15). Of course, it should be noted that homogeneous broadenings were not considered in these simulations, which will alleviate the strictness of the matching condition (vide infra).

The efficiency of the exchange of Zeeman order between two spins (A and B) is truncated in the presence of a third spin (C), if it has a stronger dipolar coupling to one of the spins (B) and is, at the same time, as well at the R^2 condition (Figure S16b). The effect of truncation is, however, diminished in case of deviation from the rotational resonance condition between C and B (Figure S16c and (d)), or the number of equivalent spins B is increased (Figure S17).

The number of spins to exchange magnetization does not have a strong effect on the build-up rates (compare Figure S17 (a) and (c) with (b) and (d), respectively). Instead, the build-up rate of the longitudinal magnetization of the inverted spins depends mainly on the distance to the source of exchanging magnetization, this is shown in Figure S18 (the effect of T_{2ZQ} will be discussed later).

A series of simulations were done to examine whether discrimination between spins within a cluster from spins outside this cluster is in principle feasible with this approach (Figure S18). The used distances were chosen to roughly reproduce the experimental curves, these values, however, are not intended to have any real physical meaning, as relaxation was not considered. The simulations reproduce three groups of spins with the same isotropic chemical shifts which are at the rotational resonance condition among each other. All spins are dipolar coupled according to their geometrical properties. As the initial condition the two smaller spin groups are inverted. Due to exchange of Zeeman order, the magnetization of the inverted spins grow, while of the magnetization of the main group decreases. Clearly distinct behavior is observed as a function of the mean distance among groups.

The effect of T_{2ZQ} on the exchange of magnetization for a pair of spins at the rotational resonance condition is shown in Figure S19. The simulated curves are in perfect agreement with the analytical expression given by Levitt[20]. When the rate of decoherence, $(T_{2ZQ})^{-1}$, is larger than the dipolar coupling, the magnetization exchange becomes slower with shorter T_{2ZQ}. As already pointed out, in this system single quantum ^{89}Y transverse relaxation times (T_2) are dominated by the paramagnetic relaxation enhancement due to gadolinium ions. Gadolinium is known to have relative (compared to other paramagnetic metal ions) long electronic relaxation times[21], which in turn results in a significant shortening of the T_2 of the surrounding nuclei. Estimation of T_{2ZQ} from addition of the individual single quantum decay rates is only a valid approximation if the source of relaxation is uncorrelated[22]. In these samples with only minor gadolinium doping, the differences in the local field fluctuations affecting two close yttrium nuclei will be minimal. Therefore, we can expect much longer T_{2ZQ} for close pairs and in the following simulations we will use a T_{2ZQ} of 1 ms as the lowest limit. In Figure S20 and S21Figure S21 (and Figure 4 of the main document) we explore different scenarios of distance, relaxation and R^2 offsets. As expected, shorter relaxation times alleviate the strictness of the R^2 matching condition. The curves evidence how different combinations of conditions can lead to similar behavior of magnetization exchange and that attributing the experimental results to a specific R^2 condition would likely be meaningless. At the same time, it becomes clear that the polarization buildup curves are very sensitive to changes in the mean conditions and therefore, should carry valuable conformational information. For instance, formation of oxygen vacancies cluster will cause a change in the mean dipolar coupling strength among $^{[6]}$Y and $^{[7]}$Y sites, which would lead to faster polarization transfer.

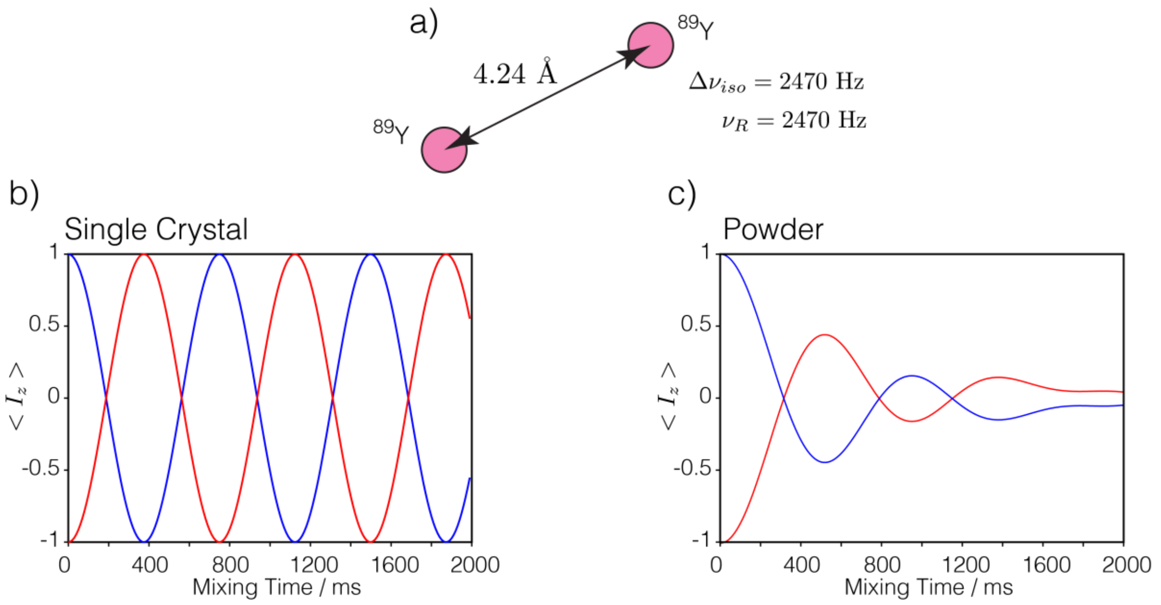

Figure S13. MAS NMR simulations of ^{89}Y-^{89}Y rotational resonance correlation for a single pair of ^{89}Y spins (a) in a single crystal (b) and in a polycrystalline sample (c) with 233 orientations. The spinning speed ν_R was set to match the difference in isotropic chemical shift of both spins. The curves (b) and (c) show the evolution of the longitudinal magnetization of both spins. As the initial conditions the magnetization of both spins was set to the same magnitude but opposite sign.

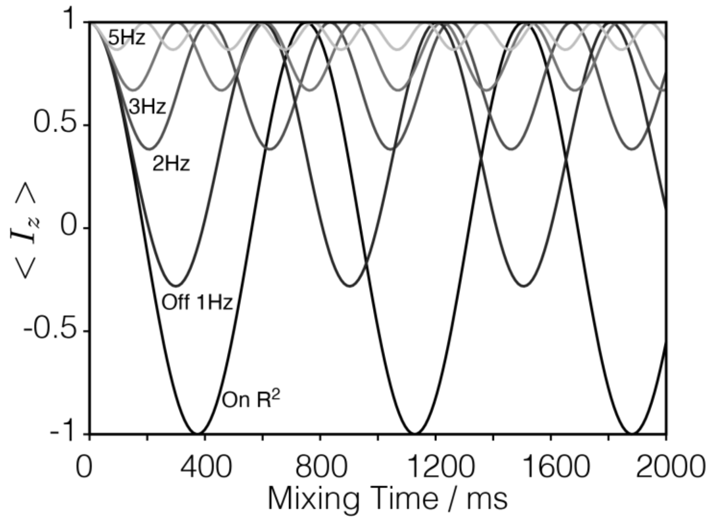

Figure S14. MAS NMR simulations of ^{89}Y-^{89}Y rotational resonance correlation for the same system shown in Figure S13 (a). The difference in isotropic chemical shift was held constant to 2470 Hz and the spinning speed was varied from 2470 (on R2) to 2475 Hz.

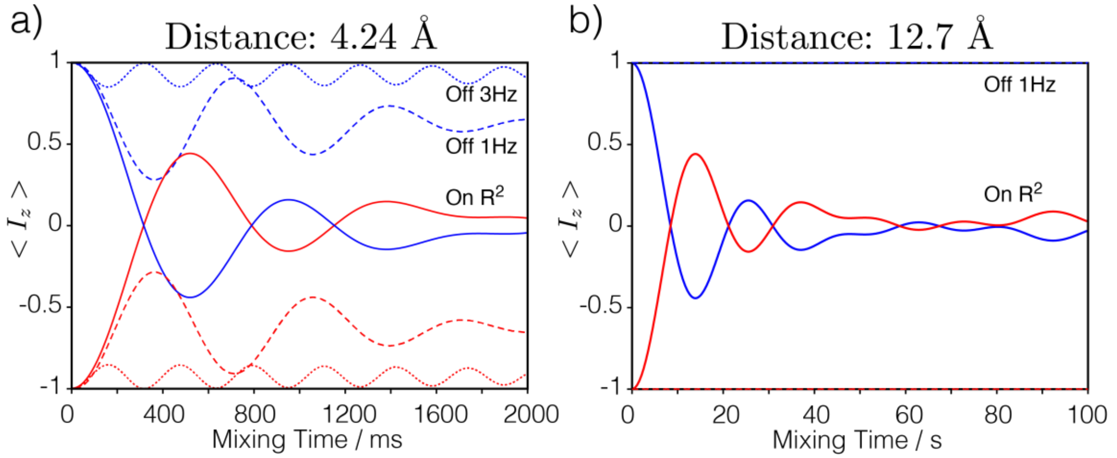

Figure S15. MAS NMR simulations of ^{89}Y-^{89}Y rotational resonance correlation for a single pair of ^{89}Y spins after averaging over 233 orientations and at a distance of 4.24 Å (a) and 12.7 Å (b). The difference in isotropic chemical shift was held constant at 2470 Hz, the spinning speed was placed exactly at the rotational resonance condition (solid lines), 1 Hz off (dashed lines) and 3 Hz off (dotted lines).

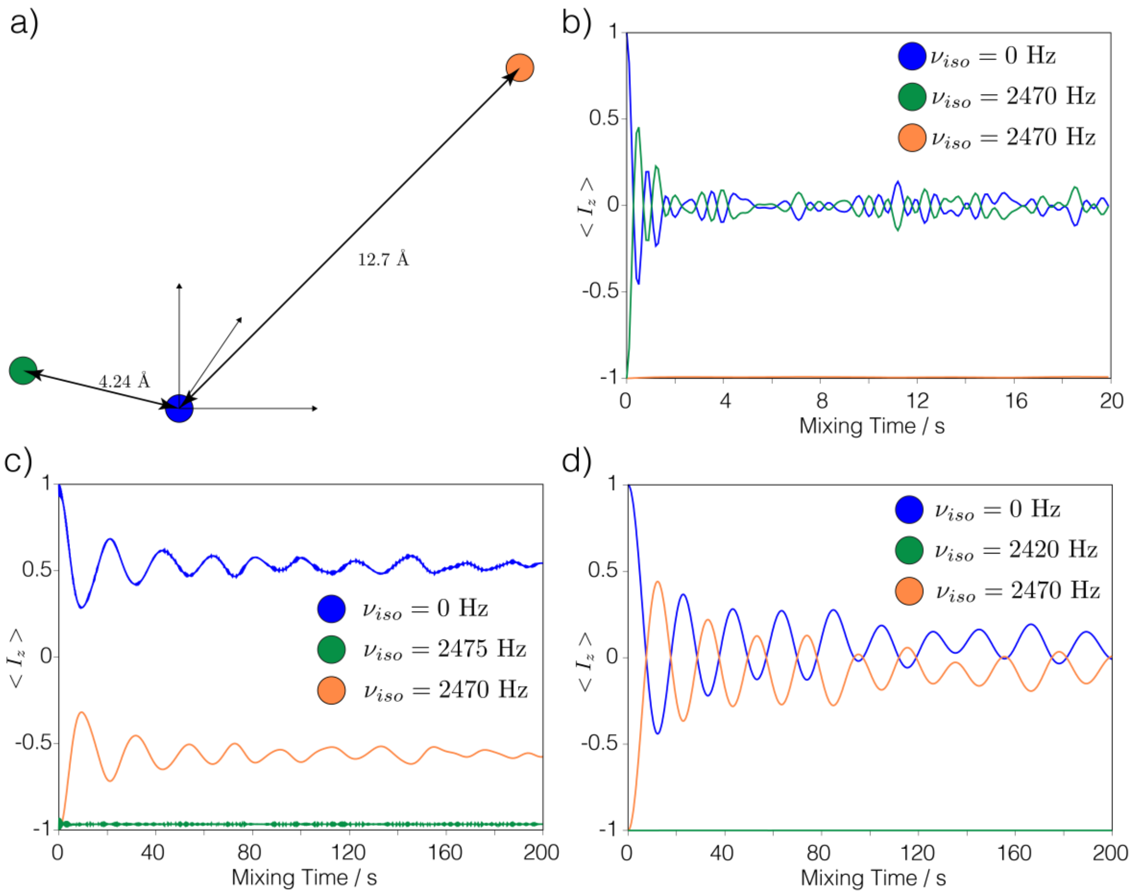

Figure S16. MAS NMR simulations of ^{89}Y-^{89}Y rotational resonance correlation for the three-spin system shown in (a) after averaging over 233 orientations. As the initial condition the magnetization of the green and orange spins was inverted with respect to the blue spin. The spinning speed ν_R was set to 2470 Hz. The blue and orange spins were held at the rotational resonance condition, the isotropic chemical shift of the green spin was varied as given in the figure legend.

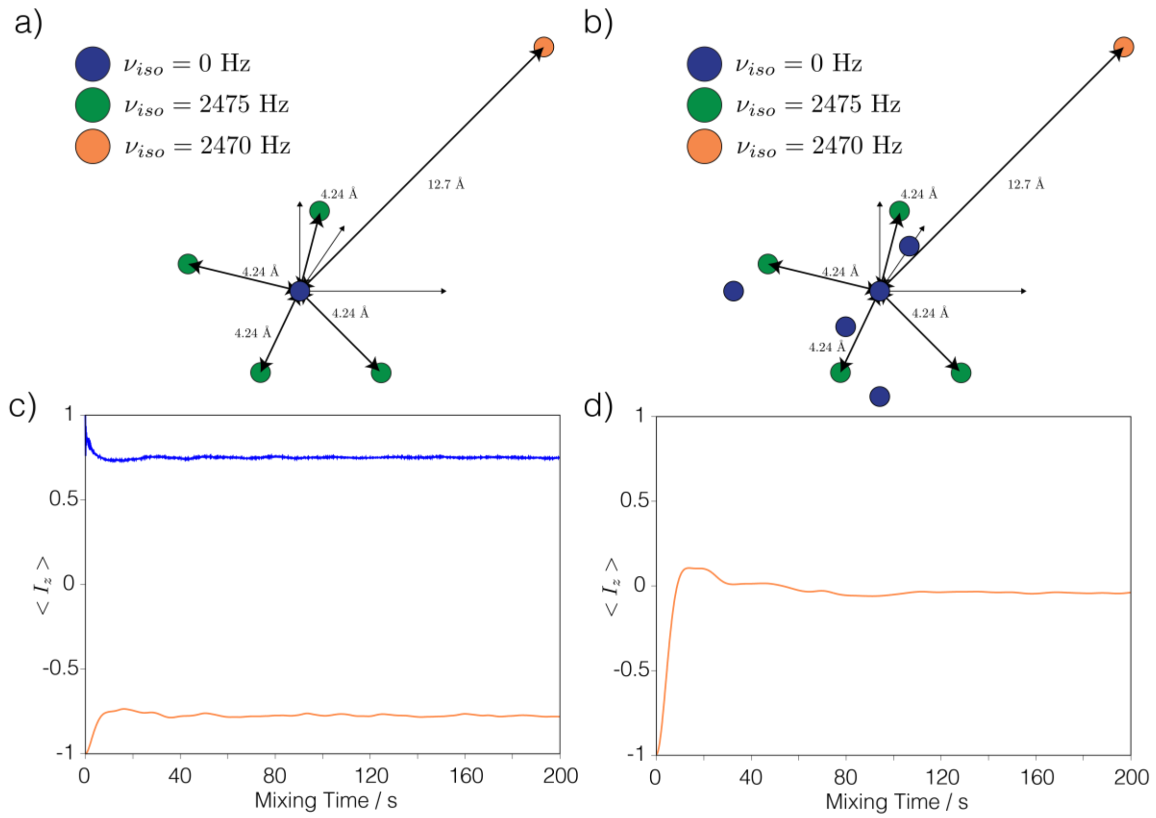

Figure S17. MAS NMR simulations of ^{89}Y-^{89}Y rotational resonance correlations in (c) and (d) for the spin systems shown in (a) and (b), respectively, after averaging over 233 orientations. As the initial condition the magnetization of the green and orange spins was inverted with respect to the blue spins. None of the additional blue spins in (b) were placed closer to the orange spin, compared to the blue spin already used in (a). The spinning speed ν_R was set to 2470 Hz to perfectly match the rotational resonance condition between the blue and orange spins.

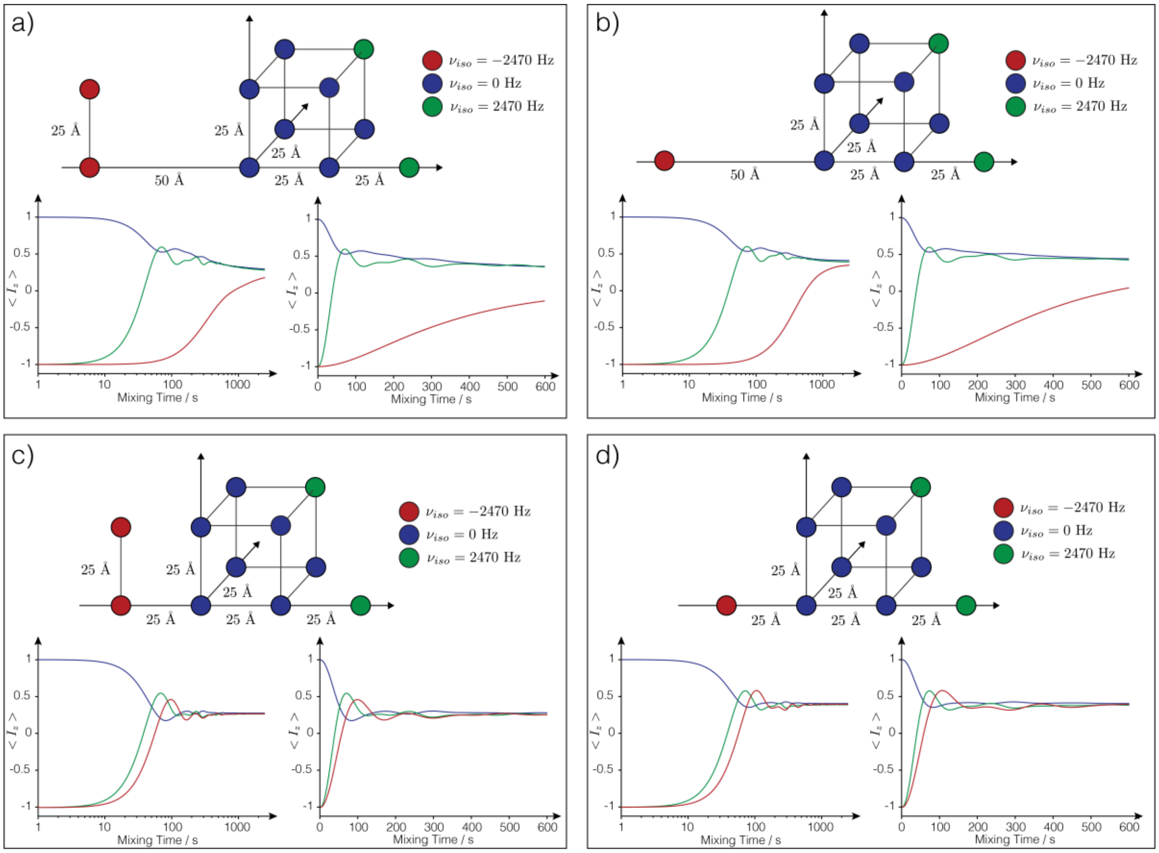

Figure S18. MAS NMR simulations of ^{89}Y-^{89}Y rotational resonance correlation using the spin-systems shown on top of each graph where every sphere represents an ^{89}Y spin and all spins are coupled via through space dipolar coupling. An average over 144 different orientations was considered. The spinning speed ν_R was set to 2470 Hz. The three spin groups (blue, green and red) are under the rotational resonance condition among each other. In (a) and (b) red and green spins have clearly distinct distances to the bath of blue spins and represent a scenario with formation of nanodomains. In figures (c) and (d) the red spins are no longer at a larger distance. In (b) and (d) only one distant nucleus was used. Each dataset is shown in a logarithmic (left) and in a linear (right) scale.

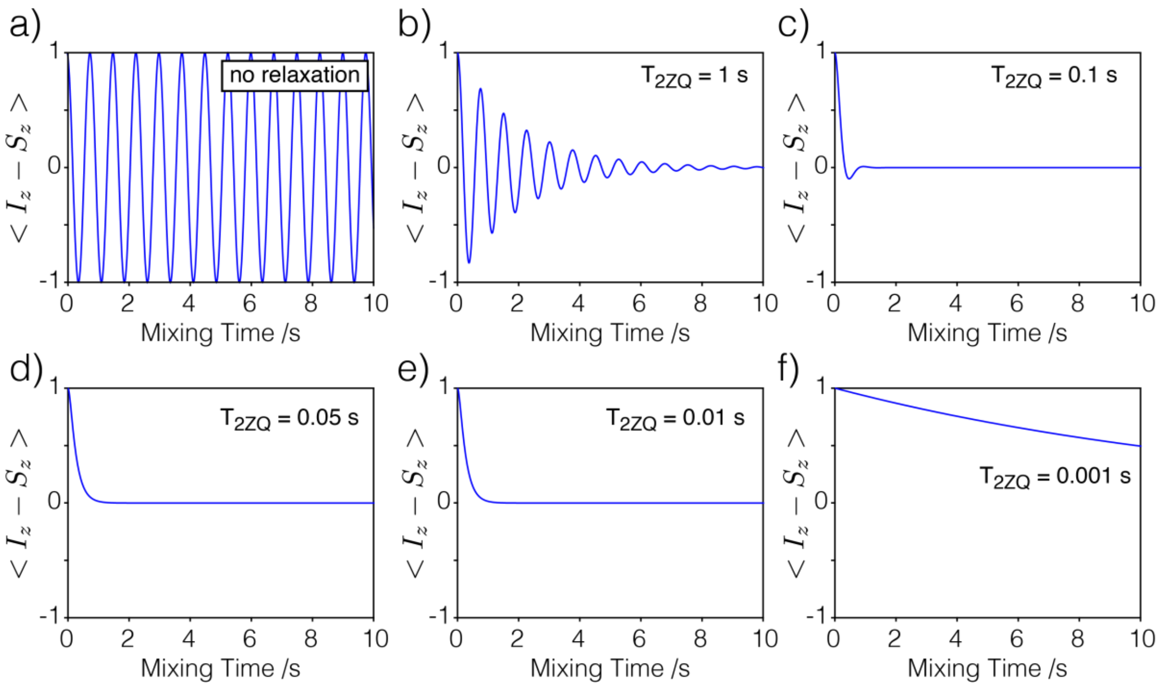

Figure S19: MAS NMR single crystal simulations of ^{89}Y-^{89}Y rotational resonance correlation for the same system shown in Figure S13 with dipolar coupling strength of approximately 4 Hz (a) with decreasing T_{2ZQ} from (a) to (f).

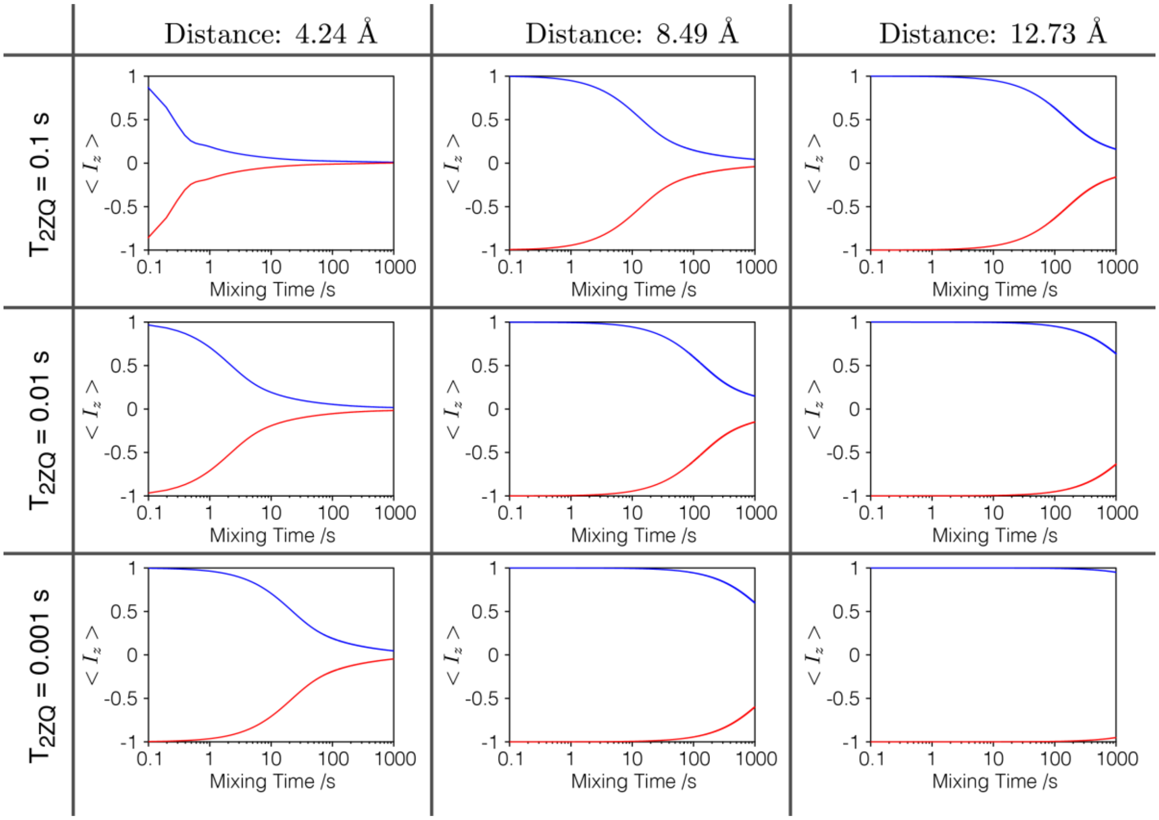

Figure S20. MAS NMR simulations of ^{89}Y-^{89}Y rotational resonance correlation for a single pair of ^{89}Y spins after averaging over 233 orientations for varying distances of 4.24, 8.49 and 12.7 Å and relaxation times T_{2ZQ} of 1, 10 and 100 ms.

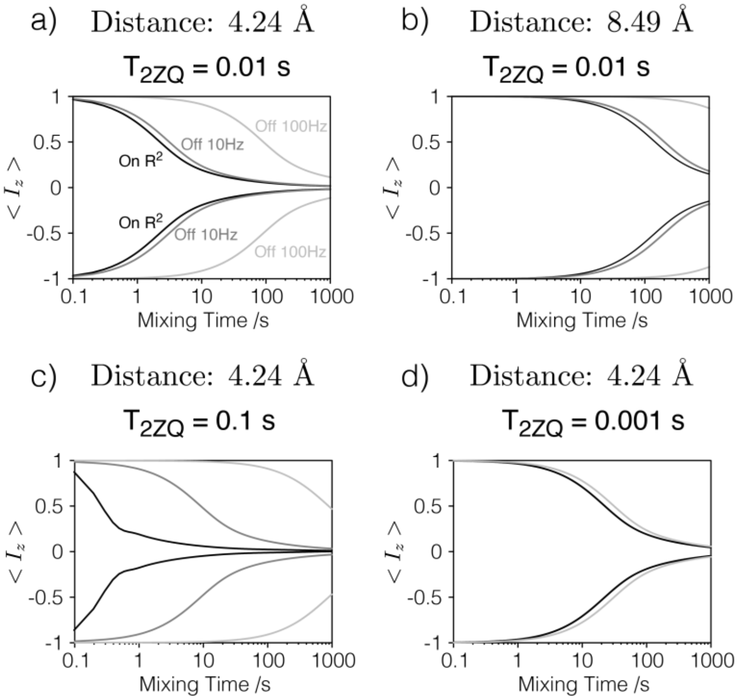

Figure S21. MAS NMR simulations of ^{89}Y-^{89}Y rotational resonance correlation for a single pair of ^{89}Y spins after averaging over 233 orientations at a distance of 4.24 Å (a, c and d) and 8.49 Å (b) and relaxation times T_{2ZQ} of 1 ms (d), 10 ms (a and b) and 100 ms (c). For the black lines the R^2 condition was matched exactly, an offset of 10 Hz is shown with the grey lines and an offset of 100 Hz with the light grey lines.

REFERENCES

(1) Yavo, N.; Nissenbaum, A.; Wachtel, E.; Shaul, T.-E.; Mendelson, O.; Kimmel, G.; Kim, S.; Lubomirsky, I.; Yeheskel, O. Rapid Sintering Protocol Produces Dense Ceria-Based Ceramics. *J. Am. Ceram. Soc.* **2018**, *101* (11), 4968–4975. https://doi.org/10.1111/jace.15743.

(2) Wallenberg, R.; Withers, R.; Bevan, D. J. M.; Thompson, J. G.; Barlow, P.; Hyde, B. G. The Fluorite-Related "Solid Solutions" of CeO_2-Y_2O_3 I: A Re-Examination by Electron Microscopy and Diffraction. *J. Less Common Met.* **1989**, *156* (1–2), 1–16. https://doi.org/10.1016/0022-5088(89)90402-5.

(3) Coduri, M.; Scavini, M.; Allieta, M.; Brunelli, M.; Ferrero, C. Defect Structure of Y-Doped Ceria on Different Length Scales. *Chem. Mater.* **2013**, *25* (21), 4278–4289. https://doi.org/10.1021/cm402359d.

(4) Merwin, L. .; Sebald, A. The First ^{89}Y CP-MAS Spectra. *J. Magn. Reson.* **1990**, *88* (1), 167–171. https://doi.org/10.1016/0022-2364(90)90121-O.

(5) Markley, J. L.; Horsley, W. J.; Klein, M. P. Spin-Lattice Relaxation Measurements in Slowly Relaxing Complex Spectra. *J. Chem. Phys.* **1971**, *55* (7), 3604–3605. https://doi.org/10.1063/1.1676626.

(6) PhySy Ltd. RMN, Version 1.8.6. (www.physyapps.com, Grandview Heights, OH 43212).

(7) Massiot, D.; Fayon, F.; Capron, M.; King, I.; Le Calvé, S.; Alonso, B.; Durand, J.-O.; Bujoli, B.; Gan, Z.; Hoatson, G. Modelling One- and Two-Dimensional Solid-State NMR Spectra. *Magn. Reson. Chem.* **2002**, *40* (1), 70–76. https://doi.org/10.1002/mrc.984.

(8) Lowe, I. J.; Tse, D. Nuclear Spin-Lattice Relaxation via Paramagnetic Centers. *Phys. Rev.* **1968**, *166* (2), 279–291. https://doi.org/10.1103/PhysRev.166.279.

(9) Jardón-Álvarez, D.; Reuveni, G.; Harchol, A.; Leskes, M. Enabling Natural Abundance ^{17}O Solid-State NMR by Direct Polarization from Paramagnetic Metal Ions. *J. Phys. Chem. Lett.* **2020**, *11* (14), 5439–5445. https://doi.org/10.1021/acs.jpclett.0c01527.

(10) Maekawa, H.; Kawata, K.; Xiong, Y. P.; Sakai, N.; Yokokawa, H. Quantification of Local Oxygen Defects around Yttrium Ions for Yttria-Doped Ceria–Zirconia Ternary System. *Solid State Ionics* **2009**, *180* (4–5), 314–319. https://doi.org/10.1016/j.ssi.2009.01.015.

(11) Florian, P.; Gervais, M.; Douy, A.; Massiot, D.; Coutures, J.-P. A Multi-Nuclear Multiple-Field Nuclear Magnetic Resonance Study of the $Y_2O_3-Al_2O_3$ Phase Diagram. *J. Phys. Chem. B* **2001**, *105* (2), 379–391. https://doi.org/10.1021/jp0008851.

(12) Kim, N.; Stebbins, J. F. Vacancy and Cation Distribution in Yttria-Doped Ceria: An ^{89}Y and ^{17}O MAS NMR Study. *Chem. Mater.* **2007**, *19* (23), 5742–5747. https://doi.org/10.1021/cm0715388.

(13) Harazono, T.; Watanabe, T. Chemical Shift, Chemical Shift Anisotropy, and Spin-Lattice Relaxation Time in ^{89}Y-MAS and -Static NMR of Yttrium Compounds. *Bull. Chem. Soc. Jpn.* **1997**, *70* (10), 2383–2388. https://doi.org/10.1246/bcsj.70.2383.

(14) Rakhmatullin, R. M.; Aminov, L. K.; Kurkin, I. N.; Böttcher, R.; Pöppl, A.; Avila-Paredes, H.; Kim, S.; Sen, S. Electron Paramagnetic Resonance Linewidth Narrowing of Gd3+ Ions in Y-Doped Ceria Nanocrystals with Decreasing Crystallite Size. *J. Chem. Phys.* **2009**, *131* (12), 124515. https://doi.org/10.1063/1.3225487.

(15) Hope, M. A.; Stevanato, G.; Halat, D. M.; Grey, C. P.; Emsley, L. Endogenous ^{17}O DNP NMR of Gd-Doped CeO_2. In *Magnetic Resonance and Magnetic Phenomena in Chemical and Biological Physics VI International School for Young Scientists*; 2020.

(16) Bertini, I.; Luchinat, C.; Parigi, G.; Ravera, E. *NMR of Paramagnetic Molecules*, 2nd ed.; Elsevier: Boston, 2017.

(17) Deguchi, H.; Yoshida, H.; Inagaki, T.; Horiuchi, M. EXAFS Study of Doped Ceria Using Multiple Data Set Fit. *Solid State Ionics* **2005**, *176* (23–24), 1817–1825. https://doi.org/10.1016/j.ssi.2005.04.043.

(18) Caravatti, P.; Bodenhausen, G.; Ernst, R. . Selective Pulse Experiments in High-Resolution Solid State NMR. *J. Magn. Reson.* **1983**, *55* (1), 88–103. https://doi.org/10.1016/0022-2364(83)90279-2.

(19) Veshtort, M.; Griffin, R. G. SPINEVOLUTION: A Powerful Tool for the Simulation of Solid and Liquid State NMR Experiments. *J. Magn. Reson.* **2006**, *178* (2), 248–282. https://doi.org/10.1016/j.jmr.2005.07.018.

(20) Levitt, M. H.; Raleigh, D. P.; Creuzet, F.; Griffin, R. G. Theory and Simulations of Homonuclear Spin Pair Systems in Rotating Solids. *J. Chem. Phys.* **1990**, *92* (11), 6347–6364. https://doi.org/10.1063/1.458314.

(21) Pintacuda, G.; John, M.; Su, X.-C.; Otting, G. NMR Structure Determination of Protein−Ligand Complexes by Lanthanide Labeling. *Acc. Chem. Res.* **2007**, *40* (3),

206–212. https://doi.org/10.1021/ar050087z.

(22) Kubo, A.; McDowell, C. A. Spectral Spin Diffusion in Polycrystalline Solids under Magic-Angle Spinning. *J. Chem. Soc. Faraday Trans. 1 Phys. Chem. Condens. Phases* **1988**, *84* (11), 3713. https://doi.org/10.1039/f19888403713.
